# Burden of heart failure attributable to chronic kidney disease in older adults (1990–2021): an analysis from the global burden of disease study

**DOI:** 10.3389/fpubh.2025.1606719

**Published:** 2025-06-18

**Authors:** Wenli Liu, Lin Huang, Yaohua Shen, Lingling Xu, Wenhua Gu, Zhaoyu Lu

**Affiliations:** ^1^The Second Clinical College of Guangzhou University of Chinese Medicine, Guangzhou, China; ^2^Department of Cardiology, Guangdong Provincial People’s Hospital, Southern Medical University, Guangzhou, China; ^3^Department of Nephrology, The Second Affiliated Hospital of Guangzhou University of Chinese Medicine, Guangzhou, China

**Keywords:** heart failure, chronic kidney disease, disease burden, trends, health inequalities

## Abstract

**Background:**

Heart failure (HF) is a critical global health issue, with chronic kidney disease (CKD) as a significant contributing factor. Both primarily affect older adults, with prevalence rising substantially after age 60. This study examined global trends and disparities in CKD-associated HF among older adults from 1990 to 2021.

**Methods:**

Utilizing data from the Global Burden of Disease (GBD) 2021, the study analyzed the prevalence and years lived with disability (YLDs) of CKD-associated HF. Joinpoint regression assessed trends from 1990 to 2021 globally, regionally, and nationally. Health inequity analysis, including the slope index of inequality and health inequality concentration index, evaluated disparities across countries.

**Results:**

From 1990 to 2021, the prevalence and YLDs of CKD-associated HF increased globally, with an average annual percentage change (AAPC) of 2.21% [95% confidence interval (CI), 2.17–2.25] and 2.20% (95% CI, 2.16–2.24), respectively. Males exhibited higher prevalence and YLDs but demonstrated a slower increase than females. The low-SDI region exhibited the highest burden, while the high-SDI region showed an unfavorable increase. Socioeconomic disparities were decreased but persisted. From 1990 to 2021, the inequality slope index for prevalence decreased from 143.66 (95% CI, 167.68–119.65) to 114.12 (95% CI, 151.59–76.65), whereas the health inequality concentration index improved from −0.21 (95% CI, −0.30 to −0.12) to −0.07 (95% CI, −0.14 to 0) for prevalence.

**Conclusion:**

The global burden of CKD-associated HF has increased substantially, with persistent disparities across gender and SDI levels. Strengthening preventive measures and implementing effective interventions are essential to addressing this escalating health challenge.

## Introduction

Heart failure (HF) is a complex clinical syndrome that affects multiple organ systems, impacting over 67 million individuals globally, with its prevalence and Years Lived with Disability (YLDs) rates exhibiting an age-related increase, particularly escalating after 60 years of age (1). It has emerged as a significant public health concern due to its high prevalence and mortality, effects on functional capacity, reduced quality of life, and substantial healthcare costs (2). Among the established risk factors for HF, chronic kidney disease (CKD) plays a particularly critical role. Affecting more than 10% of the global population, over 800 million individuals, CKD is projected to become the fifth leading cause of life expectancy reduction worldwide by 2040 (3, 4).

HF and CKD exhibit a complex, interrelated pathophysiology and clinical presentation. Notably, CKD contributes to HF development and accelerates its progression, with patients experiencing moderate to severe renal impairment facing a threefold higher risk of HF compared to those with normal renal function (2, 5). Among patients with end-stage renal disease (ESRD), up to 36% present with congestive heart failure at dialysis initiation, a prevalence 12 to 36 times higher than in the general population (6). It was reported that the presence of HF in CKD patients exacerbates clinical outcomes, leading to diminished quality of life, increased hospitalization rates, and higher mortality (7, 8). In older adults, HF-CKD comorbidity is frequently associated with frailty, more severe HF, and an elevated risk of other complications (9). The coexistence of HF and CKD imposes a substantial economic burden on healthcare systems and society as a whole (10).

A comprehensive assessment of CKD-related HF is essential to develop interventions that consider diverse socioeconomic contexts and healthcare resources, optimize management strategies, and reduce health disparities. The epidemiology of HF varied significantly across geography, age groups, and genders (11). However, CKD-associated HF, a substantial contributor to cardiovascular disease in older populations, remained inadequately studied in terms of its global, regional, and national trends and burden. Utilizing the Global Burden of Disease (GBD) database from 1990 to 2021, this study aimed to analyze the prevalence and YLDs of CKD-associated HF in older adults, examining their burden and trends at global, regional, and national levels and cross-country inequalities. By assessing the burden of CKD-associated HF, we aimed to provide evidence-based support for personalized disease prevention and management programs, and contribute to the scientific basis for promoting more effective and equitable health policies.

## Methods

### Study design

This study retrospectively analyzed population-based, repeated cross-sectional data from the GBD 2021, obtained from the Global Health Data Exchange database. Covering 371 diseases and injuries across 204 countries and territories from 1990 to 2021, the GBD 2021 employed standardized analytical methods tailored to each cause, integrating vital statistics, health surveys, and disease registries to systematically estimate disease burden by location, year, age, and sex (12, 13). In accordance with Executive Order No. 7724 (May 16, 2012) and Resolution No. 510 (April 7, 2016), the GBD studies utilize publicly available, de-identified data, thus negating the requirement for ethics committee approval.

### Case definition and data collection

The GBD study defines HF impairment utilizing structured diagnostic criteria, such as the Framingham or European Society of Cardiology (ESC) guidelines. The Framingham approach necessitates either two major criteria or one major and two minor criteria, while the ESC method emphasizes typical signs and symptoms resulting from cardiac structural and/or functional abnormalities. HF is categorized into four severity levels: treated, mild, moderate, and severe. The GBD study encompasses individuals with American College of Cardiology (ACC)/American Heart Association (AHA) stage C and beyond, including both symptomatic patients and those diagnosed with HF but asymptomatic. For GBD 2021, each etiology of stage 5 CKD is allocated a disability weight based on the severity of HF (mild, moderate, or severe). CKD is characterized by the progressive deterioration of kidney function, assessed through estimated glomerular filtration rate (eGFR) and the urinary albumin-to-creatinine ratio (ACR). The International Classification of Diseases, 10th Revision (ICD-10) designates CKD codes N18.1-N18.9 (12, 13).

This investigation focused on older adults aged 60 and older, the demographic most susceptible to CKD-associated HF. The research evaluated the global, regional, and national specific burden of CKD-related HF on this population by analyzing prevalence, YLDs, and corresponding rates extracted from the GBD 2021 dataset. Employing GBD methodology, these rates were reported per 100,000 individuals, accompanied by 95% uncertainty intervals (UIs) for each metric. CKD-associated HF indicators were stratified by sex, five HF severity levels, five socio-demographic index (SDI) levels, 21 regions defined by epidemiological and geographical factors, and 204 countries and territories. Each HF severity level has a specific disability weight, which quantifies health loss on a scale from 0 (no health loss) to 1 (equivalent to death). Additional details regarding severity levels and their associated disability weights are available on the GBD website (12, 13). Countries and territories were categorized into five SDI quintiles: low, low-middle, middle, high-middle, and high. The SDI serve as a composite metric that captures socioeconomic factors affecting health outcomes. This index includes three key elements: fertility rates among individuals under 25, time-adjusted per capita income, and mean educational attainment for those aged 15 and older. On this scale, a score of 0 represents the extreme values of the highest fertility rate, lowest per capita income and educational attainment (12, 13).

### Trend analysis

Joinpoint regression analysis was utilized to assess trends in CKD-associated HF from 1990 to 2021. This methodology initially posits a linear trend in disease impact throughout the study period. An inflection point is introduced to denote a shift in this trend. Statistical significance is assessed by comparing the joinpoint model to the null model using a permutation test, with the joinpoint retained if deemed statistically significant. The method incorporates the Bonferroni correction to address multiple comparisons, selecting optimal joinpoints from the Monte Carlo permutation test (14).

This methodology was employed to analyze linear and non-linear trends by examining their inflection points and associated weights. The magnitude of change was evaluated utilizing annual percentage change (APC) and average APC (AAPC) values, accompanied by their 95% confidence intervals (CIs). The APC, a statistical metric, quantifies the rate of change between different inflection points, characterizing the slope for each specified time interval. The AAPC functions as an analytical tool that summarizes the overall trend across a specified period, indicating whether it increased, remained constant, or decreased. To determine the AAPC, a weighted average of APC values from the joinpoint model was calculated, with each APC value weighted based on the duration of its corresponding time period. AAPCs were determined for the period from 1990 to 2021. Statistical significance of the AAPC is established when the interval excludes zero (14).

### Cross-country inequality analysis

Two key metrics were employed to assess the impact of CKD-associated HF across different countries and territories: the slope index of inequality and the health inequality concentration index for absolute and relative disparities, respectively. These World Health Organization-endorsed measures provide a comprehensive assessment of health inequalities (15).

The slope index of inequality was determined through regression analysis. The age-standardized rates of prevalence and YLDs of CKD-associated HF at the country level served as dependent variables, while the independent variable was a relative social status scale, defined by the midpoint of cumulative population intervals ranked by the SDI. To address heteroskedasticity, a robust regression model utilizing repeated reweighted least squares with a Huber weighting function was utilized. The health inequality concentration index was computed based on the Lorenz concentration curve, which illustrates the cumulative distribution relationship between the SDI-ordered population and the CKD-associated HF burden. Numerical integration of the area under the curve was utilized to calculate the index (16). All statistical analyses were conducted using R (version 4.2.2; Posit PBC, Boston, MA, USA).

## Results

### Global CKD-associated HF burden and trends

**Figure 1:**
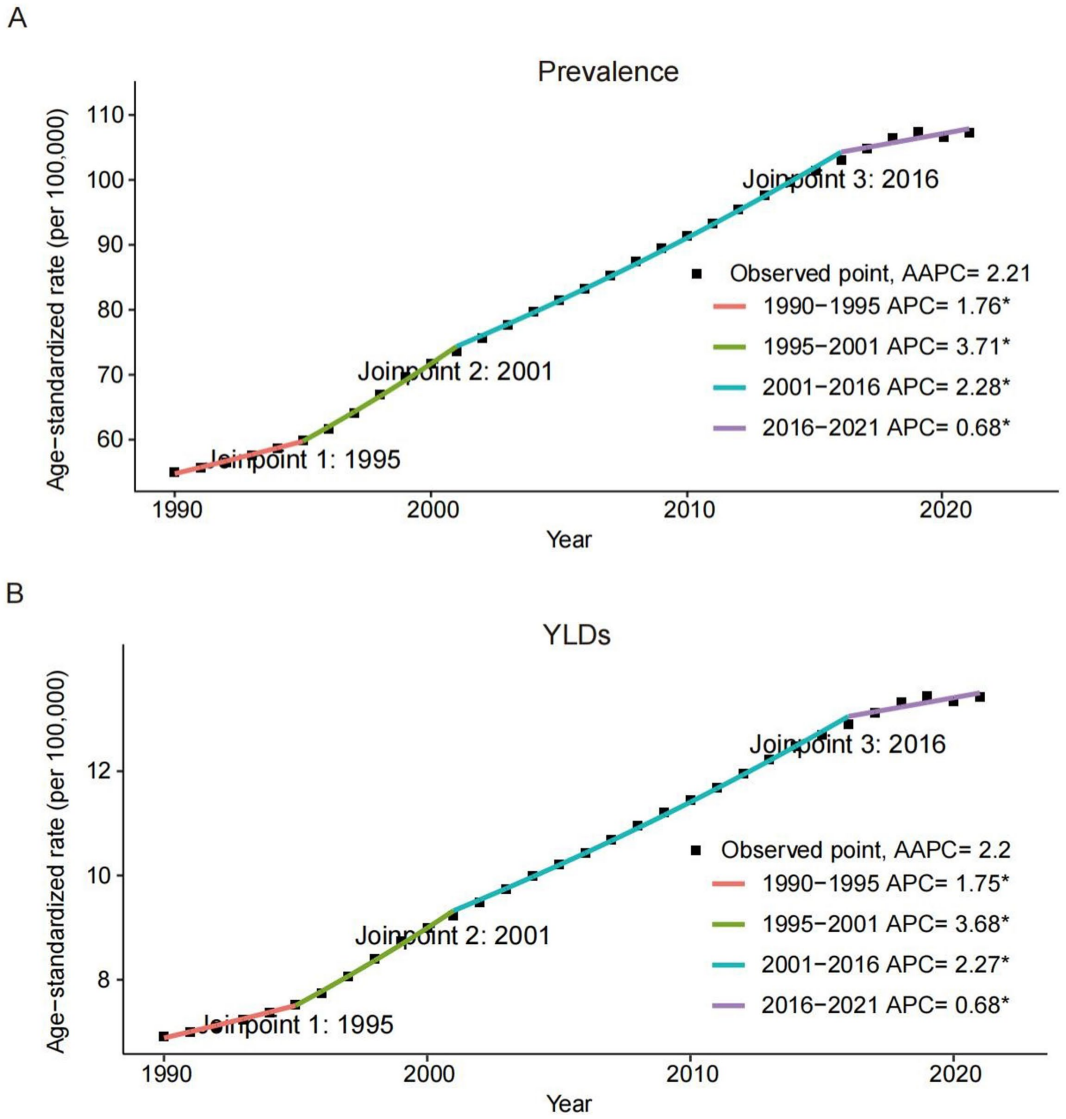
Joinpoint regression analysis of the global prevalence **(A)** and YLDs **(B)** rates of heart failure attributable to chronic kidney diseases among older adults from 1990 to 2021. APC, annual percentage change; AAPC, average annual percentage change; YLDs, years lived with disability.

**Figure 2:**
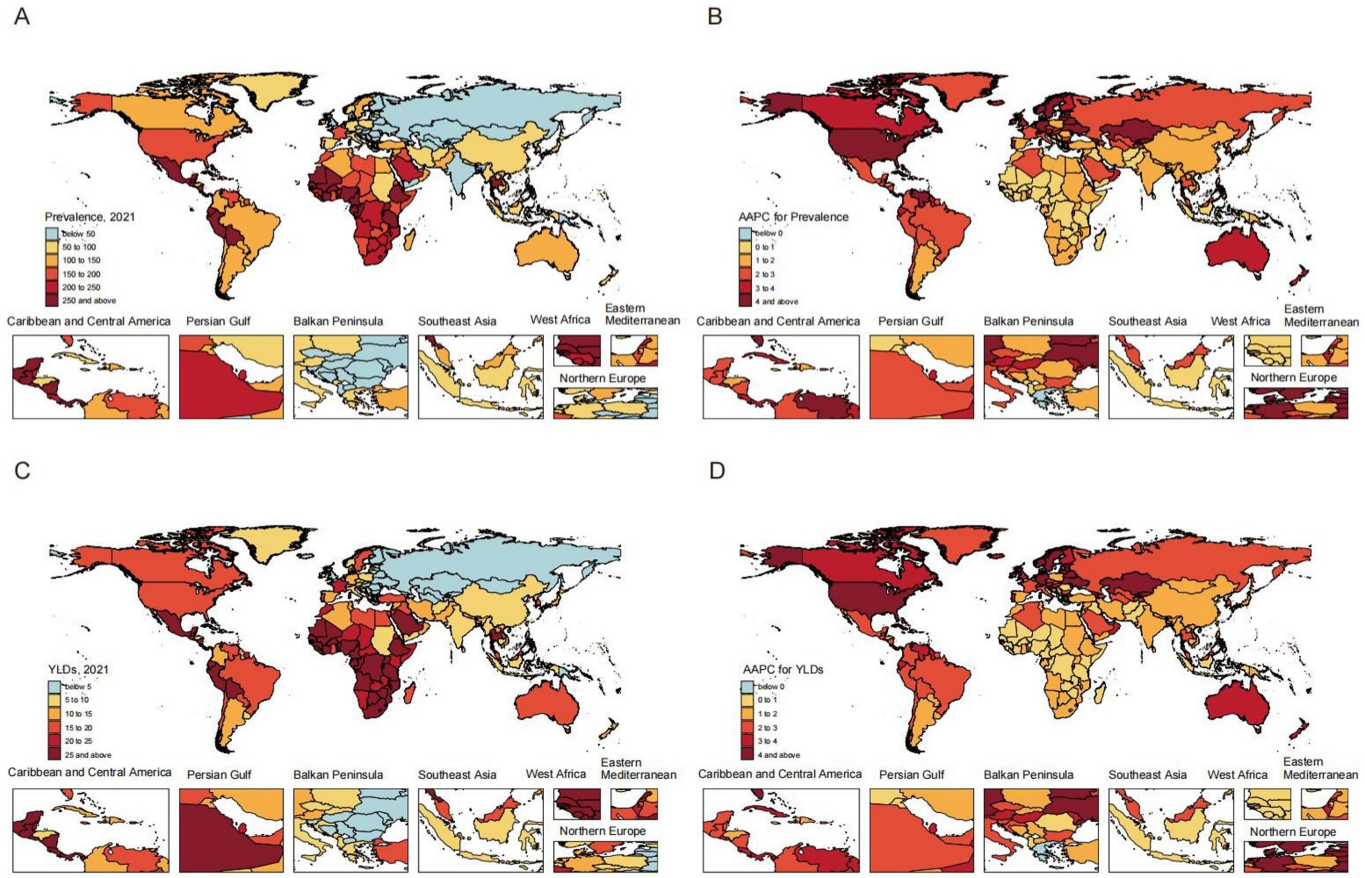
Global prevalence **(A)** and YLDs **(C)** rates of heart failure attributable to chronic kidney diseases among adults in 2021; Alterations in the overall trend of prevalence **(B)** and YLDs **(D)** rates of heart failure attributable to chronic kidney diseases among adults. AAPC, average annual percentage change; YLDs, years lived with disability.

Tables 1, 2, along with Figures 1, 2, illustrated the burden and trends in prevalence and YLDs rates for CKD-associated HF among individuals aged 60 and older from 1990 to 2021.

**Table 1:** Prevalence of heart failure attributable to chronic kidney disease among older adults and corresponding AAPCs from 1990 to 2021 at the global level.

	Case, 1990	Prevalence, 1990	Case, 2021	Prevalence, 2021	AAPC, 1990–2021	*p* value
Global	234779.15 (149784.57–353100.76)	55.02 (34.88–82.78)	1123837.92 (685895.57–1737896.47)	107.23 (65.36–165.71)	2.21 (2.17 to 2.25)	<0.001
By sex						
Male	115189.81 (73038.99–174446.29)	66.75 (41.87–101.01)	532987.96 (322055.05–827203.53)	118.72 (71.44–184.09)	1.88 (1.85 to 1.9)	<0.001
Female	119589.33 (76570.39–179259.47)	47.92 (30.55–71.93)	590849.96 (362289.45–913093.89)	99.78 (61.23–154.1)	2.42 (2.36 to 2.47)	<0.001
By severity						
Treated heart failure	86277.18 (53962.1–130876.24)	20.22 (12.58–30.68)	412987.23 (249956.77–645585.53)	39.41 (23.81–61.52)	2.21 (2.17 to 2.25)	<0.001
Mild heart failure	43703.91 (24934.71–69275.81)	10.24 (5.83–16.31)	209213.82 (114868.4–340118.63)	19.96 (10.93–32.48)	2.21 (2.17 to 2.25)	<0.001
Moderate heart failure	28317.29 (16369.55–46745.78)	6.63 (3.83–10.93)	135548.03 (76586.53–228167.1)	12.93 (7.3–21.74)	2.21 (2.17 to 2.25)	<0.001
Severe heart failure	76480.76 (47390.17–116931.41)	17.92 (11.04–27.44)	366088.84 (221242.05–576330.43)	34.93 (21.07–54.98)	2.21 (2.17 to 2.25)	<0.001
By SDI						
High SDI	60395.32 (37144.62–91472.25)	42.49 (26.13–64.46)	367793.35 (221406.65–571725.95)	119.32 (72.23–184.97)	3.43 (3.35 to 3.5)	<0.001
High-middleSDI	37026.44 (23809.21–55196.09)	33.88 (21.61–50.75)	170386.45 (104222.29–263998.7)	67.93 (41.48–105.22)	2.31 (2.23 to 2.38)	<0.001
Middle SDI	68129.34 (44257.46–101280.06)	71.03 (45.87–105.3)	356332.84 (220909.93–548958.39)	116.57 (72.06–179.34)	1.64 (1.6 to 1.68)	<0.001
Low-middle SDI	39654.86 (25246.76–59914.83)	71.55 (45.24–107.84)	145099.68 (88253.23–226167.76)	97.31 (58.85–151.38)	1 (0.97 to 1.02)	<0.001
Low SDI	29357.43 (15902.07–49119.39)	152.8 (82.72–253.19)	83353.09 (45162.53–138528.49)	186.77 (101.18–308.71)	0.64 (0.6 to 0.67)	<0.001

AAPC, average annual percentage change; SDI, sociodemographic index.

**Table 2:** YLDs of heart failure attributable to chronic kidney disease among older adults and corresponding AAPCs from 1990 to 2021 at the global level.

	Number, 1990	YLDs, 1990	Number, 2021	YLDs, 2021	AAPC, 1990–2021	*p* value
Global	29663.05 (16165.52–50371.7)	6.92 (3.77–11.7)	140949.75 (74773.09–243444.58)	13.43 (7.12–23.18)	2.2 (2.16 to 2.24)	<0.001
By sex						
Male	14533.93 (7873.55–24900.58)	8.35 (4.53–14.22)	66889.1 (35108.33–115779.84)	14.84 (7.78–25.64)	1.87 (1.85 to 1.9)	<0.001
Female	15129.12 (8251.51–25415.39)	6.04 (3.3–10.13)	74060.65 (39547.91–127549.82)	12.51 (6.68–21.54)	2.39 (2.35 to 2.44)	<0.001
By severity						
Treated heart failure	12476.46 (6714.82–21206.77)	2.91 (1.57–4.93)	59281.71 (31206.06–102303.49)	5.65 (2.97–9.75)	2.2 (2.16 to 2.24)	<0.001
Mild heart failure	1793.33 (822.04–3248.62)	0.42 (0.19–0.76)	8540.05 (3843.05–15656.72)	0.81 (0.37–1.49)	2.2 (2.16 to 2.24)	<0.001
Moderate heart failure	2010.91 (998.51–3686.85)	0.47 (0.23–0.86)	9577.55 (4754.27–17806.52)	0.91 (0.45–1.69)	2.2 (2.16 to 2.24)	<0.001
Severe heart failure	13382.35 (7110.53–23052.62)	3.12 (1.66–5.36)	63550.44 (33091.46–110973.98)	6.05 (3.15–10.57)	2.2 (2.16 to 2.24)	<0.001
By SDI						
High SDI	7693.98 (4088.14–12935.72)	5.41 (2.88–9.08)	46140.46 (24167.18–79593.33)	15.01 (7.87–25.88)	3.4 (3.33 to 3.47)	<0.001
High-middle SDI	4732.92 (2586.58–7861.26)	4.31 (2.35–7.15)	21509.75 (11442.62–36994.34)	8.56 (4.56–14.74)	2.28 (2.21 to 2.35)	<0.001
Middle SDI	8598.08 (4760.71–14411.37)	8.87 (4.93–14.79)	44671.23 (23972.7–76735.6)	14.56 (7.81–24.98)	1.64 (1.6 to 1.68)	<0.001
Low-middle SDI	4968.23 (2682.37–8494.01)	8.88 (4.82–15.13)	18170.64 (9521.09–31754.67)	12.11 (6.35–21.11)	1.01 (0.98 to 1.03)	<0.001
Low SDI	3642.45 (1752.99–6778.37)	18.75 (9.1–34.53)	10348.12 (4946.82–19252.73)	22.98 (11.1–42.41)	0.65 (0.61 to 0.67)	<0.001

YLDs, years lived with disability; AAPC, average annual percentage change; SDI, sociodemographic index.

Globally, both prevalence and YLDs rates for CKD-associated HF demonstrated an increasing trend from 1990 to 2021, with the AAPC of 2.21 (95% CI, 2.17–2.25; *p* < 0.001) for prevalence and 2.20 (95% CI, 2.16–2.24; *p* < 0.001) for YLDs. Regarding gender, males exhibited a higher burden in prevalence and YLDs compared to females, but a slower increase from 1990 to 2021. The AAPCs for females were 2.42 (95% CI, 2.36–2.47; *p* < 0.001) for prevalence and 2.39 (95% CI, 2.35–2.44; *p* < 0.001) for YLDs. In contrast, males had lower AAPCs of 1.88 (95% CI, 1.85–1.90; *p* < 0.001) for prevalence and 1.87 (95% CI, 1.85–1.90; *p* < 0.001) for YLDs. Across severity levels, the trends of CKD-related HF in prevalence and YLDs showed no significant difference, consistent with global trends.

Among SDI groups, the low-SDI group exhibited the highest prevalence of 186.77 (95% UI, 101.18–308.71) and YLDs of 22.98 (95% UI, 11.1–42.41), followed by the high-SDI group. The high-middle-SDI group displayed the lowest prevalence of 67.93 (95% UI, 41.48–105.22) and YLDs of 8.56 (95% UI, 4.56–14.74). Adverse trends in prevalence and YLDs were observed, with more rapid increases correlating with higher SDI levels. The high-SDI group showed the greatest increases, with AAPCs of 3.43 (95% CI: 3.35–3.50) for prevalence and 3.40 (95% CI: 3.33–3.47) for YLDs. In contrast, the low-SDI group had the smallest increases, with AAPCs of 0.64 (95% CI: 0.60–0.67) and 0.65 (95% CI: 0.61–0.67) for prevalence and YLDs, respectively.

### CKD-associated HF burden and trends by region

The burden and trends of CKD-associated HF exhibited significant regional variations. Western Sub-Saharan Africa demonstrated the highest rates in 2021, with a prevalence of 493.71 (95% UI, 275.73–783.84) and a YLDs of 60.64 (95% UI, 30.47–109.11). Conversely, Eastern Europe displayed the lowest prevalence and YLDs, at 16.79 (95% UI, 9.22–28.13) and 2.17 (95% UI, 1.03–3.99), respectively. From 1990 to 2021, High-income North America experienced the most substantial increases in prevalence and YLDs, with AAPCs of 4.38 (95% CI, 4.31–4.47) and 4.28 (95% CI, 4.21–4.34), respectively. In contrast, Central Sub-Saharan Africa exhibited the smallest increases, with AAPCs of 0.55 (95% CI, 0.52–0.58) for prevalence and 0.54 (95% CI, 0.52–0.56) for YLDs (Supplementary Tables S1, S2).

### CKD-associated HF burden and trends by country/territory

At the country/territory level, Nigeria exhibited the highest prevalence of 775.85 (95% UI, 442.28–1196.81) and YLDs of 95.14 (95% UI, 48.64–166.46) in 2021. Conversely, Ukraine demonstrated the lowest prevalence of 3.66 (95% UI, 1.9–6.39) and YLDs of 0.47 (95% UI, 0.22–0.89). From 1990 to 2021, Armenia showed the most substantial increases in prevalence [AAPC: 10.48 (95% CI, 10.19–10.8)] and YLDs [AAPC: 10.44 (95% CI, 10.15–10.75)]. In contrast, Kuwait had the least pronounced increases in prevalence [AAPC: 0.27 (95% CI, 0.16–0.38)] and YLDs [AAPC: 0.28 (95% CI, 0.18–0.38)]. Furthermore, Greece exhibited no significant increase and a trend toward decrease, with an AAPC of −0.01 (95% CI, −0.23 to 0.19) in prevalence and an AAPC of −0.02 (95% CI, −0.24 to 0.19) in YLDs (Supplementary Tables S3, S4).

### Cross-country inequalities

**Figure 3:**
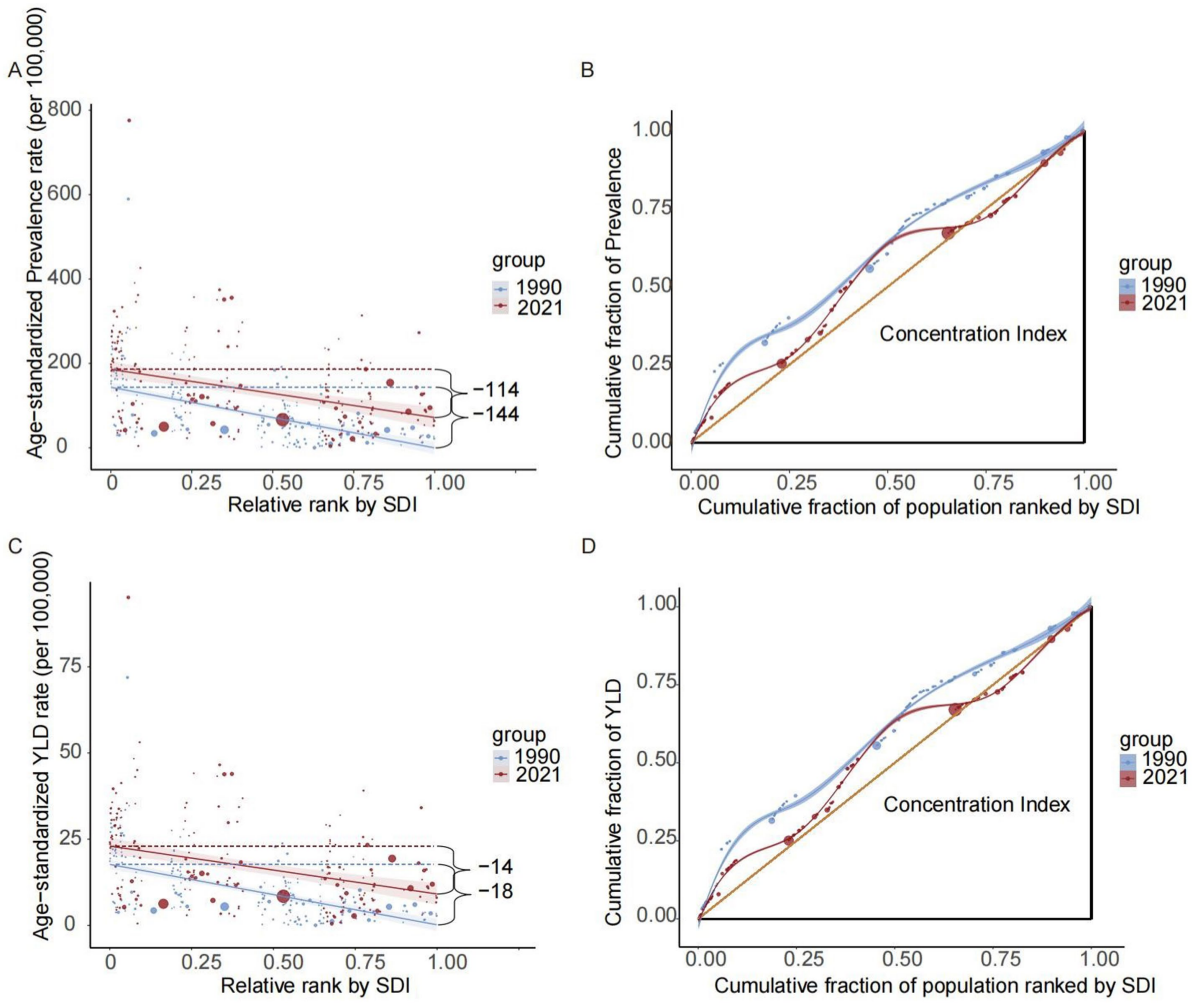
Disparities in the burden of heart failure attributable to chronic kidney diseases among older adults by development status. Health disparity regression curves of prevalence **(A)** and YLDs **(C)**; Health disparity concentration curves of prevalence **(B)** and YLDs **(D)**. YLDs, years lived with disability; SDI, sociodemographic index.

Our analysis of 204 countries and territories revealed substantial SDI-related disparities in the burden of CKD-associated HF. This burden was predominantly observed in regions with lower SDIs; however, these inequalities have decreased over time. The slope index of absolute inequality showed that age-standardized prevalence rate among the countries and territories with the lowest and highest SDIs was 143.66 (95% CI, 167.68–119.65) in 1990, reducing to 114.12 (95% CI, 151.59–76.65) by 2021. Similarly, the age-standardized YLDs rate difference between the lowest and highest SDI countries/territories decreased from 17.52 (95% CI, 20.48–14.56) in 1990 to 13.89 (95% CI, 18.53–9.24) in 2021. The health inequality concentration index was −0.21 (95% CI, −0.30 to −0.12) (*p* < 0.05) for prevalence and −0.20 (95% CI, −0.29 to −0.11) (*p* < 0.05) for YLDs rate in 1990. These values changed to −0.07 (95% CI, −0.14 to 0) (*p* < 0.05) for prevalence and −0.06 (95% CI, −0.14 to 0.01) (*p* < 0.05) for YLDs rate in 2021, indicating that the burden remained disproportionately concentrated in less-developed regions (Figure 3; Table 3).

**Table 3:** Inequalities in age-standardized prevalence and YLD rates of heart failure attributable to chronic kidney disease among older adults, by sociodemographic index.

Health inequality metrics	Slope index of inequality (absolute gradient)		Health concentration index (relative gradient)	
	Value	95% CI	value	95% CI
Prevalence				
1900	−143.66	(−167.68 to −119.65)	−0.21	(−0.30 to −0.12)
2021	−114.12	(−151.59 to −76.65)	−0.07	(−0.14 to 0)
YLDs				
1900	−17.52	(−20.48 to −14.56)	−0.2	(−0.29 to −0.11)
2021	−13.89	(−18.53 to −9.24)	−0.06	(−0.13 to 0.01)

YLDs, years lived with disability.

## Discussion

This study investigated the global burden and trends of CKD-associated HF among individuals aged 60 and older from 1990 to 2021. The principal findings were as follows: (1) The global prevalence and YLDs of CKD-associated HF demonstrated a significant increase. (2) Males exhibited a higher burden in both prevalence and YLDs compared to females in 2021. (3) Significant SDI-related disparities were identified, with the burden more pronounced in regions with lower SDIs. The low-SDI region demonstrated the highest prevalence and YLDs; particularly, Western Sub-Saharan Africa demonstrated the highest burden in 2021. (4) The high-middle-SDI region displayed the lowest prevalence and YLDs, while the high-SDI region demonstrated the most substantial increases from 1990 to 2021, presenting additional complexities for future public health initiatives.

The global increase in prevalence and YLDs of CKD-associated HF from 1990 to 2021 corresponds with the worldwide trend of population aging. Research demonstrated that both CKD and HF exhibited higher prevalence in older populations, potentially leading to their increased co-occurrence as the older demographic expands (1, 17). This trend also reflected global shifts in health patterns, particularly the evolving disease spectrum, which has intensified multimorbidity in older adults. It was reported that conditions such as diabetes, hypertension, and frailty frequently coexist and interact, with shared mechanisms—chronic inflammation, metabolic dysfunction, and oxidative stress—driving the increased risk of both CKD and HF (18–20). Moreover, as life expectancy continues to increase, prolonged exposure to shared risk factors for CKD and HF, such as hypertension, diabetes, and obesity, elevates the risk of synergistic disease progression (19). The escalating global prevalence of diabetes, hypertension, and obesity further underscored the growing challenge of CKD-associated HF. Notably, type 2 diabetes (T2D) and hypertension, in particular, were the primary etiological factors for ESRD, accounting for over 70% of cases, and the obesity epidemic served as a major contributor of rising T2D incidence (21, 22). The interplay between obesity, T2D, and hypertension complicates efforts to elucidate the specific contributions of each factor to the pathogenesis of CKD-associated HF. Targeted interventions to manage hypertension, diabetes, and obesity may help to mitigate the rising burden of CKD-associated HF.

The observed trends can be partially attributed to advancements in diagnostic capabilities and evolving disease classifications, especially in high-SDI region, where there is increased access to healthcare resources and ongoing improvements in healthcare technology. Improved CKD staging methods, including refined eGFR formulas and standardized albuminuria measurements, have enhanced detection accuracy (23). Concurrently, HF diagnostics evolved from primarily clinical assessments to incorporating sensitive biomarkers, such as Natriuretic Peptide (BNP) and N-Terminal Pro-BNP, as well as advanced imaging techniques, facilitating earlier identification, particularly in older adults with comorbidities (24). The increased recognition of HF with preserved ejection fraction (HFpEF), prevalent in CKD patients but historically underdiagnosed, has substantially expanded the diagnosed HF population (25). Besides, contemporary HF treatments have demonstrated a 28% reduction in mortality compared to conventional approaches (26). Numerous new pharmacological agents, such as angiotensin-converting enzyme (ACE) inhibitors, *β*-receptor antagonists, and sodium-glucose cotransporter-2 (SGLT2) inhibitors, along with treatments like implantable devices and heart transplantation, have decelerated the disease progression, improved cardiac function, and enhanced survival rates (27). However, while these advances extend life expectancy, they also increase the overall disease burden of CKD-associated HF. Prolonged survival in patients with both CKD and HF, particularly among older adults, results in greater morbidity, higher hospital admissions, and an increased need for long-term care, further straining healthcare systems (8).

In 2021, males exhibited a higher burden of CKD-associated HF compared to females, a disparity driven by multiple factors. Research indicated that males are more significantly exposed to hyperlipidemia, overweight, diabetes, hypertension, and other risk factors shared between CKD and cardiovascular disease (28, 29). Unhealthy lifestyle behaviors in males may constitute another significant contributing factor. Males demonstrated a higher propensity for tobacco use, excessive alcohol consumption, and insufficient physical activity (28). Moreover, studies noted that kidney function deteriorated more rapidly in males than females (30), likely attributed to unhealthy lifestyles and hormonal effects. For instance, testosterone has been implicated in accelerating kidney damage, while estrogen appears to have protective effects (31, 32). Females, in contrast, generally experienced a more favorable prognosis in HF, with lower hospitalization and mortality rates (33), possibly due to differences in pathophysiological mechanisms between sexes, such as females exhibiting lower mitochondrial oxidative damage, higher energy metabolism, greater resistance to fibroinflammatory responses, and estrogenic protection (34, 35).

Beyond physiological and lifestyle factors, healthcare disparities may contribute to the observed sex differences in CKD-associated HF burden. Firstly, the diagnostic criteria for CKD and HF were predominantly developed using male cohorts, whereas females often exhibit different clinical presentations, potentially leading to underdiagnosis or delayed diagnosis. Secondly, the underrepresentation of females in HF clinical trials has resulted in insufficient female-specific evidence. Drug dosages and device criteria from male-dominated studies may reduce treatment efficacy and survival in females, thereby may increasing mortality risk (33, 36). Furthermore, awareness of CKD is lower among females compared to males, which may delay their pursuit of medical consultation and intervention (37). Traditional cultural and social systems further restrict females’ healthcare access, with inequalities exacerbated by resource shortages and high treatment costs. In low-SDI regions, in particular, females have limited economic power and depend on male family members for healthcare funding (38, 39).

Our study revealed a higher burden of CKD-associated HF in regions with lower SDI, with the highest prevalence and YLDs observed in the low-SDI region. Research indicated that individuals in lower SDIs were at increased risk of CKD or HF compared to higher SDI regions (40, 41). The disparity in disease burden between low and high-middle SDI regions may be attributed to inequities in access to prevention, diagnostic, and treatment services in the low-SDI region. Specifically, the insufficiency of pathology and laboratory diagnostic services, paucity of healthcare professionals, and limited coverage of health services in the low-SDI region exacerbated health management challenges (42, 43). The density of nephrologists was 80 and 10 times lower in low- and middle-income countries than in high-income countries (44). Research found that four essential cardiovascular medications including *β*-blockers, aspirin, statins, and ACE inhibitors were unavailable or unaffordable in substantial portions of low-income countries (45). Notably, the low-SDI region exhibited the highest YLD burden, may largely attributed to inadequate dialysis or renal replacement therapy (RRT) and delayed in medical care. A study highlighted that only 32% of low-income countries provide dialysis to more than half of ESRD patients, compared to 98% in high-income countries (44). The 2023 global kidney health atlas also underscored the significant treatment gap, with much lower RRT access in low- and middle-income countries. In addition, disease awareness and health education are integral to shaping individual health outcomes, guiding clinical decision-making, and influencing healthcare utilization (46). Individuals with lower socioeconomic and educational levels are less likely to recognize their CKD status due to limited access to quality healthcare resources (47).

Besides, the low-SDI region experienced disproportionately severe environmental pollution and sanitation issues. Study showed that the low-SDI region suffered the highest mortality rates from air pollution and inadequate hand hygiene facilities (48). Despite being among the most affected by climate change, largely due to heavy reliance on fossil fuels and delayed transitions to clean energy, only 18.7 to 26.7% of countries in Central and South America, Asia, and Africa addressed the climate–health link in their 2018–2020 UN General Assembly statements (49, 50). Soil pollution also exhibited notable regional disparities, with heavy metal contamination particularly acute in developing countries such as India and those in Central Africa (51). Exposure to environmental pollutants—including contaminated soil, water, and air—is a well-established risk factor for chronic conditions such as hypertension, diabetes, CKD, and HF (52). Mechanistically, prolonged exposure to these pollutants may trigger oxidative stress, systemic inflammation, and autonomic dysfunction, ultimately impairing cardiovascular and renal systems and accelerating the progression of CKD and HF (53–55).

The systemic healthcare disparities may partially explain the highest disease burden observed in West Sub-Saharan Africa in our study. Countries with disparate healthcare coverage models showed substantial differences in CKD-associated HF burden. According to the Global Monitoring Report on Universal Health Coverage, Sub-Saharan Africa had the lowest coverage of essential health services worldwide (56). In this region, most nations lack comprehensive social welfare systems or health insurance infrastructure, with inadequate coverage that rendered secondary and tertiary CKD care financially catastrophic and made RTT largely inaccessible (39). A study reported that in Sub-Saharan Africa, 96% of adults and 95% of children with ESRD requiring dialysis died or were presumed dead due to the unaffordability of treatment (57). Nigeria exemplified these challenges as Sub-Saharan Africa’s most populous nation and largest economy, recording the highest CKD-associated HF burden globally at the country/territory level. Limited insurance coverage combined with high out-of-pocket costs forced patients to discontinue chronic disease management (58). Additionally, the emigration of healthcare professionals was a critical concern, with over 12,000 Nigerian physicians practicing abroad, contributing to severe shortages in specialties such as cardiology and nephrology (59). Conversely, high-income Asia-Pacific countries, including Brunei, Japan, South Korea, and Singapore, have established universal health insurance or public-private healthcare models that ensured accessibility to ESRD treatment for most patients (39).

Our findings indicated that the burden of CKD-associated HF in the high-SDI region was ranked second to low-SDI regions. Research demonstrated that while HF incidence has stabilized or declined in high-income countries, its burden continued to increase, potentially driven by aging populations, increased risk factors, efficacy of novel therapies, and improved survival rates (2). Furthermore, the trend among different SDI groups increased more rapidly as the SDI levels increased during the period from 1990 to 2021. Multiple factors contributed to this seemingly paradoxical phenomenon. Social development has reduced mortality from infectious diseases, while population aging, prolonged chronic disease duration, and behavioral risk factors such as physical inactivity, smoking, and alcohol consumption increase the risk of CKD or HF (60, 61). Additionally, advancements in medical technology and updated diagnostic criteria have also amplified the disease burden statistically. These factors may elucidate why high-income North America experienced the most substantial increases in prevalence and YLDs from 1990 to 2021. These findings necessitate that policymakers reconsider the assumption that development inherently improves health, and balance health risks and social progress through preventive interventions, timely treatment, and disease surveillance.

In response to the growing global burden of CKD-associated HF, comprehensive preventive measures are urgently needed. Primarily, promoting healthy lifestyles is essential for preventing and mitigating disease burden. Encouraging balanced nutrition, regular physical activity, smoking cessation, and alcohol moderation can reduce metabolic diseases such as obesity, hypertension, and diabetes (62). Besides, research has shown that dietary optimization and regular exercise play a crucial role in improving renal function and lowering HF risk (63, 64). The World Health Organization (WHO) is committed to reducing healthcare disparities and promoting health equity. To alleviate the burden of CKD-associated HF, regions with lower SDI should focus on enhancing medical training, improving health infrastructure, expanding healthcare coverage, and increasing access to CKD- and HF-related medications through international collaboration. Moreover, achieving environmental sustainability is critical for advancing the United Nations’ 2030 Sustainable Development Goals and enhancing global health outcomes. Evidence suggested that strengthening environmental governance and mitigating pollution effectively prevent cardiovascular and kidney diseases (65, 66). Therefore, it is imperative for countries to balance economic growth with environmental protection, recognizing the link between environmental degradation and chronic diseases.

This research has some limitations. Firstly, our investigation was based on the GBD database, which, while methodologically robust and reliable, is ultimately restricted by the quality of accessible data. Secondly, in certain locations, particularly those with lower SDI, GBD data is often limited or insufficient, predominantly relying on statistical modeling (12, 13). This constraint is likely to affect the analysis and potentially introduce bias into burden estimates. However, the GBD framework addressed this limitation by incorporating UIs in all estimates, which capture uncertainties throughout the estimation process, especially in data-sparse regions. In our secondary analysis using the joinpoint method, we further incorporated UIs while assuming non-constant variance (12, 13). The acquisition of additional high-quality datasets will be essential for enhancing future research endeavors. Thirdly, the GBD database lacked subnational data for most countries, limiting our ability to conduct such analyses within individual countries. Future updates with improved granularity would enable more comprehensive assessments of health inequalities within countries. Finally, despite the GBD study’s robust methodological adjustments to account for COVID-19 disruptions in data completeness and disease detection, some uncertainty in the 2020–2021 estimates remained.

## Conclusion

In conclusion, this research demonstrated that the global burden of CKD-associated HF in older adults has generally increased from 1990 to 2021, with significant disparities persisting across genders and SDI levels. Regions with lower socioeconomic development continued to bear a disproportionately higher burden. The findings from this population-based investigation will inform evidence-driven resource allocation for effective prevention and intervention strategies targeting CKD-associated HF. The increasing impact of this condition necessitates prioritization in global and national health agendas, with policymakers urged to enhance investments in preventive medicine and implement interventions with demonstrated efficacy.

## Data Availability

Publicly available datasets were analyzed in this study. This data can be found here: https://www.healthdata.org/research-analysis/gbd.

## References

[1] Ran J Zhou P Wang J Zhao X Huang Y Zhou Q. Global, regional, and national burden of heart failure and its underlying causes, 1990-2021: results from the global burden of disease study 2021. Biomark Res. (2025) 13:16. doi: 10.1186/S40364-025-00728-8, PMID: 39849627 PMC11755835

[2] Khan MS Shahid I Bennis A Rakisheva A Metra M Butler J. Global epidemiology of heart failure. Nat Rev Cardiol. (2024) 21:717–34. doi: 10.1038/S41569-024-01046-6 38926611

[3] Kovesdy CP. Epidemiology of chronic kidney disease: an update 2022. Kidney Int Suppl. (2011) 12:7–11. doi: 10.1016/J.Kisu.2021.11.003 PMC9073222 35529086

[4] Foreman KJ Marquez N Dolgert A Fukutaki K Fullman N McGaughey M. Forecasting life expectancy, years of life lost, and all-cause and cause-specific mortality for 250 causes of death: reference and alternative scenarios for 2016-40 for 195 countries and territories. Lancet. (2018) 392:2052–90. doi: 10.1016/S0140-6736(18)31694-5, PMID: 30340847 PMC6227505

[5] Kottgen A Russell SD Loehr LR Crainiceanu CM Rosamond WD Chang PP. Reduced kidney function as a risk factor for incident heart failure: the atherosclerosis risk in communities (Aric) study. J Am Soc Nephrol. (2007) 18:1307–15. doi: 10.1681/Asn.2006101159, PMID: 17344421

[6] Bhatti NK Karimi Galougahi K Paz Y Nazif T Moses JW Leon MB. Diagnosis and management of cardiovascular disease in advanced and end-stage renal disease. J Am Heart Assoc. (2016) 5:e003648. doi: 10.1161/Jaha.116.003648, PMID: 27491836 PMC5015288

[7] McNally T Tumelty E Chung I Hussain S Mookerjee S Ali MA. Investigating the relationship between frailty and quality of life in patients with heart failure and CKD (frail study). ESC Heart Fail. (2024) 11:1411–21. doi: 10.1002/Ehf2.14693 38320815 PMC11098643

[8] Bansal N Zelnick L Bhat Z Dobre M He J Lash J. Burden and outcomes of heart failure hospitalizations in adults with chronic kidney disease. J Am Coll Cardiol. (2019) 73:2691–700. doi: 10.1016/J.Jacc.2019.02.071, PMID: 31146814 PMC6590908

[9] Díez-Villanueva P Jiménez-Méndez C Pérez-Rivera Á Caballero EB López J Ortiz C. Different impact of chronic kidney disease in older patients with heart failure according to frailty. Eur J Intern Med. (2025) 132:90–6. doi: 10.1016/J.Ejim.2024.12.001 39648049

[10] Nichols GA Ustyugova A Déruaz-Luyet A O’keeffe-Rosetti M Brodovicz KG. Health care costs by type of expenditure across eGFR stages among patients with and without diabetes, cardiovascular disease, and heart failure. J Am Soc Nephrol. (2020) 31:1594–601. doi: 10.1681/Asn.2019121308, PMID: 32487562 PMC7350988

[11] Liu Z Li Z Li X Yan Y Liu J Wang J. Global trends in heart failure from 1990 to 2019: an age-period-cohort analysis from the global burden of disease study. ESC Heart Fail. (2024) 11:3264–78. doi: 10.1002/Ehf2.14915 38937863 PMC11424301

[12] GBD 2021 Diseases And Injuries Collaborators. Global incidence, prevalence, years lived with disability (YLDs), disability-adjusted life-years (DALYs), and healthy life expectancy (HALE) for 371 diseases and injuries in 204 countries and territories and 811 subnational locations, 1990-2021: a systematic analysis for the global burden of disease study 2021. Lancet. (2024) 403:2133–61. doi: 10.1016/S0140-6736(24)00757-8, PMID: 38642570 PMC11122111

[13] GBD 2021 Causes Of Death Collaborators. Global burden of 288 causes of death and life expectancy decomposition in 204 countries and territories and 811 subnational locations, 1990-2021: a systematic analysis for the global burden of disease study 2021. Lancet. (2024) 403:2100–32. doi: 10.1016/S0140-6736(24)00367-2, PMID: 38582094 PMC11126520

[14] Chen J Cao X Xu S Chen X Xie R Ye G. Global, regional, and national burden of retinoblastoma in infants and young children: findings from the global burden of disease study 1990-2021. EClinicalMedicine. (2024) 76:102860. doi: 10.1016/J.Eclinm.2024.102860, PMID: 39398496 PMC11470412

[15] World Health Organization. Handbook on health inequality monitoring, with a special focus on low- and middle-income countries. Geneva: World Health Organization (2013).

[16] Chen J Zhu Y Li L Lv J Li Z Chen X. Visual impairment burden in retinopathy of prematurity: trends, inequalities, and improvement gaps. Eur J Pediatr. (2024) 183:1891–900. doi: 10.1007/S00431-024-05450-5, PMID: 38319404

[17] Chesnaye NC Ortiz A Zoccali C Stel VS Jager KJ. The impact of population ageing on the burden of chronic kidney disease. Nat Rev Nephrol. (2024) 20:569–85. doi: 10.1038/S41581-024-00863-9, PMID: 39025992

[18] Liu P Li Y Zhang Y Mesbah SE Ji T Ma L. Frailty and hypertension in older adults: current understanding and future perspectives. Hypertens Res. (2020) 43:1352–60. doi: 10.1038/S41440-020-0510-5, PMID: 32651557

[19] Matsushita K Ballew SH Wang AY Kalyesubula R Schaeffner E Agarwal R. Epidemiology and risk of cardiovascular disease in populations with chronic kidney disease. Nat Rev Nephrol. (2022) 18:696–707. doi: 10.1038/S41581-022-00616-6, PMID: 36104509

[20] Ferrucci L Fabbri E. Inflammageing: chronic inflammation in ageing, cardiovascular disease, and frailty. Nat Rev Cardiol. (2018) 15:505–22. doi: 10.1038/S41569-018-0064-2, PMID: 30065258 PMC6146930

[21] Maric-Bilkan C. Obesity and diabetic kidney disease. Med Clin North Am. (2013) 97:59–74. doi: 10.1016/J.Mcna.2012.10.010, PMID: 23290730 PMC3539140

[22] Burnier M Damianaki A. Hypertension as cardiovascular risk factor in chronic kidney disease. Circ Res. (2023) 132:1050–63. doi: 10.1161/Circresaha.122.321762, PMID: 37053276

[23] Levin A Ahmed SB Carrero JJ Foster B Francis A Hall RK. Executive summary of the KDIGO 2024 clinical practice guideline for the evaluation and management of chronic kidney disease: known knowns and known unknowns. Kidney Int. (2024) 105:684–701. doi: 10.1016/J.Kint.2023.10.016, PMID: 38519239

[24] Mcdonagh TA Metra M Adamo M Gardner RS Baumbach A Böhm M. 2023 focused update of the 2021 esc guidelines for the diagnosis and treatment of acute and chronic heart failure: developed by the task force for the diagnosis and treatment of acute and chronic heart failure of the European Society of Cardiology (ESC) with the special contribution of the heart failure association (HFA) of the ESC. Eur J Heart Fail. (2024) 26:5–17. doi: 10.1002/Ejhf.3024, PMID: 38169072

[25] House AA Wanner C Sarnak MJ Piña IL Mcintyre CW Komenda P. Heart failure in chronic kidney disease: conclusions from a kidney disease: improving global outcomes (KDIGO) controversies conference. Kidney Int. (2019) 95:1304–17. doi: 10.1016/J.Kint.2019.02.022, PMID: 31053387

[26] Karlström P Pivodic A Dahlström U Fu M. Modern heart failure treatment is superior to conventional treatment across the left ventricular ejection Spectrum: real-life data from the Swedish heart failure registry 2013-2020. Clin Res Cardiol. (2024) 113:1355–68. doi: 10.1007/S00392-024-02498-Z, PMID: 39186181 PMC11371852

[27] Huitema AA Harkness K Malik S Suskin N Mckelvie RS. Therapies for advanced heart failure patients ineligible for heart transplantation: beyond pharmacotherapy. Can J Cardiol. (2020) 36:234–43. doi: 10.1016/J.Cjca.2019.11.012 32036865

[28] Vallianou NG Mitesh S Gkogkou A Geladari E. Chronic kidney disease and cardiovascular disease: is there any relationship. Curr Cardiol Rev. (2019) 15:55–63. doi: 10.2174/1573403X14666180711124825, PMID: 29992892 PMC6367692

[29] Pinho-Gomes A Peters S Thomson B Woodward M. Sex differences in prevalence, treatment and control of cardiovascular risk factors in England. Heart. (2020). doi: 10.1136/Heartjnl-2020-317446 32887737

[30] Melsom T Norvik JV Enoksen IT Stefansson V Mathisen UD Fuskevåg OM. Sex differences in age-related loss of kidney function. J Am Soc Nephrol. (2022) 33:1891–902. doi: 10.1681/Asn.2022030323, PMID: 35977806 PMC9528336

[31] Carrero JJ Hecking M Chesnaye NC Jager KJ. Sex and gender disparities in the epidemiology and outcomes of chronic kidney disease. Nat Rev Nephrol. (2018) 14:151–64. doi: 10.1038/Nrneph.2017.181, PMID: 29355169

[32] Brar A Markell M. Impact of gender and gender disparities in patients with kidney disease. Curr Opin Nephrol Hypertens. (2019) 28:178–82. doi: 10.1097/Mnh.0000000000000482, PMID: 30652978

[33] Qiu W Wang W Wu S Zhu Y Zheng H Feng Y. Sex differences in long-term heart failure prognosis: a comprehensive meta-analysis. Eur J Prev Cardiol. (2024) 31:2013–23. doi: 10.1093/Eurjpc/Zwae256, PMID: 39101475

[34] Regitz-Zagrosek V Kararigas G. Mechanistic pathways of sex differences in cardiovascular disease. Physiol Rev. (2017) 97:1–37. doi: 10.1152/Physrev.00021.2015, PMID: 27807199

[35] Colafella K Denton K. Sex-specific differences in hypertension and associated cardiovascular disease. Nat Rev Nephrol. (2018) 14:185–201. doi: 10.1038/Nrneph.2017.189, PMID: 29380817

[36] Rosano G Stolfo D Anderson L Abdelhamid M Adamo M Bauersachs J. Differences in presentation, diagnosis and management of heart failure in women. A scientific statement of the heart failure association of the ESC. Eur J Heart Fail. (2024) 26:1669–86. doi: 10.1002/Ejhf.3284, PMID: 38783694

[37] Hödlmoser S Winkelmayer WC Zee J Pecoits-Filho R Pisoni RL Port FK. Sex differences in chronic kidney disease awareness among us adults, 1999 to 2018. PLoS One. (2020) 15:E0243431. doi: 10.1371/Journal.Pone.0243431, PMID: 33338051 PMC7748269

[38] García GG Iyengar A Kaze F Kierans C Padilla-Altamira C Luyckx V. Sex and gender differences in chronic kidney disease and access to care around the globe. Semin Nephrol. (2022) 42:101–13. doi: 10.1016/J.Semnephrol.2022.04.001, PMID: 35718358

[39] Carrero JJ Hecking M Ulasi I Sola L Thomas B. Chronic kidney disease, gender, and access to care: a global perspective. Semin Nephrol. (2017) 37:296–308. doi: 10.1016/J.Semnephrol.2017.02.009, PMID: 28532558

[40] Zeng X Liu J Tao S Hong H Li Y Fu P. Associations between socioeconomic status and chronic kidney disease: a meta-analysis. J Epidemiol Community Health. (2018) 72:270–9. doi: 10.1136/Jech-2017-209815 29437863

[41] Conrad N Judge A Tran J Mohseni H Hedgecott D Crespillo AP. Temporal trends and patterns in heart failure incidence: a population-based study of 4 million individuals. Lancet. (2018) 391:572–80. doi: 10.1016/S0140-6736(17)32520-5, PMID: 29174292 PMC5814791

[42] Wilson ML Fleming KA Kuti MA Looi LM Lago N Ru K. Access to pathology and laboratory medicine services: a crucial gap. Lancet. (2018) 391:1927–38. doi: 10.1016/S0140-6736(18)30458-6, PMID: 29550029

[43] Crisp N Chen L. Global supply of health professionals. N Engl J Med. (2014) 370:950–7. doi: 10.1056/Nejmra1111610, PMID: 24597868

[44] Bello AK Okpechi IG Levin A Ye F Damster S Arruebo S. An update on the global disparities In kidney disease burden and care across world countries and regions. Lancet Glob Health. (2024) 12:e382–95. doi: 10.1016/S2214-109X(23)00570-3 38365413

[45] Khatib R Mckee M Shannon H Chow C Rangarajan S Teo K. Availability and affordability of cardiovascular disease medicines and their effect on use in high-income, middle-income, and low-income countries: an analysis of the PURE study data. Lancet. (2016) 387:61–9. doi: 10.1016/S0140-6736(15)00469-9, PMID: 26498706

[46] Saab MM Landers M Hegarty J. Exploring awareness and help-seeking intentions for testicular symptoms among heterosexual, gay, and bisexual men in Ireland: a qualitative descriptive study. Int J Nurs Stud. (2017) 67:41–50. doi: 10.1016/J.Ijnurstu.2016.11.016, PMID: 27915088

[47] Iroegbu C Lewis L Matura LA. An integrative review: chronic kidney disease awareness and the social determinants of health inequities. J Adv Nurs. (2022) 78:918–28. doi: 10.1111/Jan.15107, PMID: 34910316

[48] Liu Q Deng J Yan W Qin C Du M Wang Y. Burden and trends of infectious disease mortality attributed to air pollution, unsafe water, sanitation, and hygiene, and non-optimal temperature globally and in different socio-demographic index regions. Glob Health Res Policy. (2024) 9:23. doi: 10.1186/S41256-024-00366-X, PMID: 38937833 PMC11212388

[49] Romanello M Napoli CD Green C Kennard H Lampard P Scamman D. The 2023 report of the lancet countdown on health and climate change: the imperative for a health-centred response in a world facing irreversible harms. Lancet. (2023) 402:2346–94. doi: 10.1016/S0140-6736(23)01859-7, PMID: 37977174 PMC7616810

[50] Fisher S Bellinger DC Cropper ML Kumar P Binagwaho A Koudenoukpo JB. Air pollution and development in Africa: impacts on health, the economy, and human capital. Lancet Planet Health. (2021) 5:e681–8. doi: 10.1016/S2542-5196(21)00201-1, PMID: 34627472

[51] Hou D Jia X Wang L Mcgrath SP Zhu YG Hu Q. Global soil pollution by toxic metals threatens agriculture and human health. Science. (2025) 388:316–21. doi: 10.1126/Science.Adr5214, PMID: 40245139

[52] Bowe B Xie Y Li T Yan Y Xian H Al-Aly Z. The 2016 global and national burden of diabetes mellitus attributable to Pm2·5 air pollution. Lancet Planet Health. (2018) 2:e301–12. doi: 10.1016/S2542-5196(18)30140-2 30074893

[53] Scimeca M Palumbo V Giacobbi E Servadei F Casciardi S Cornella E. Impact of the environmental pollution on cardiovascular diseases: from epidemiological to molecular evidence. Heliyon. (2024) 10:e38047. doi: 10.1016/J.Heliyon.2024.E38047 39328571 PMC11425171

[54] Wang M Zhou T Song Y Li X Ma H Hu Y. Joint exposure to various ambient air pollutants and incident heart failure: a prospective analysis in UK biobank. Eur Heart J. (2021) 42:1582–91. doi: 10.1093/Eurheartj/Ehaa1031, PMID: 33527989 PMC8060055

[55] Liu G Wang ZK Wang ZY Yang DB Liu ZP Wang L. Mitochondrial permeability transition and its regulatory components are implicated in apoptosis of primary cultures of rat proximal tubular cells exposed to Lead. Arch Toxicol. (2016) 90:1193–209. doi: 10.1007/S00204-015-1547-0, PMID: 26082307

[56] World Health Organization and International Bank for Reconstruction Tracking universal health coverage: 2017 global monitoring report. World Health Organization and International Bank for Reconstruction and Development / The World Bank (2017).

[57] Ashuntantang G Osafo C Olowu WA Arogundade F Niang A Porter J. Outcomes in adults and children with end-stage kidney disease requiring dialysis In sub-Saharan Africa: a systematic review. Lancet Glob Health. (2017) 5:e408–17. doi: 10.1016/S2214-109X(17)30057-8 28229924

[58] Angell B Sanuade O Adetifa IM Okeke IN Adamu AL Aliyu MH. Population health outcomes In Nigeria compared with other west African countries, 1998-2019: a systematic analysis for the global burden of disease study. Lancet. (2022) 399:1117–29. doi: 10.1016/S0140-6736(21)02722-7, PMID: 35303469 PMC8943279

[59] Buchan J Campbell J Mccarthy C. Correction to: optimizing the contributions of nursing and midwifery workforces: #protect, #invest, #together. Hum Resour Health. (2021) 19:38. doi: 10.1186/S12960-021-00583-2, PMID: 33752684 PMC7983196

[60] Aggarwal M Bozkurt B Panjrath G Aggarwal B Ostfeld RJ Barnard ND. Lifestyle modifications for preventing and treating heart failure. J Am Coll Cardiol. (2018) 72:2391–405. doi: 10.1016/J.Jacc.2018.08.2160, PMID: 30384895

[61] Li Z Zhao H Wang J. Metabolism and chronic inflammation: the links between chronic heart failure and comorbidities. Front Cardiovasc Med. (2021) 8:650278. doi: 10.3389/Fcvm.2021.650278, PMID: 34026868 PMC8131678

[62] Rutters F Den Braver NR Lakerveld J Mackenbach JD van der Ploeg HP Griffin S. Lifestyle interventions for cardiometabolic health. Nat Med. (2024) 30:3455–67. doi: 10.1038/S41591-024-03373-0, PMID: 39604492

[63] Adachi K Uchiyama K Muraoka K Nakayama T Yasuda M Miyashita K. Home-based exercise program ameliorates renal function decline in patients with CKD stage 4. Kidney Int Rep. (2022) 7:899–903. doi: 10.1016/J.Ekir.2022.01.006 35497789 PMC9039468

[64] Ibsen DB Levitan EB Åkesson A Gigante B Wolk A. The dash diet is associated with a lower risk of heart failure: a cohort study. Eur J Prev Cardiol. (2022) 29:1114–23. doi: 10.1093/Eurjpc/Zwac003, PMID: 34983068

[65] Shrestha P Nukala SK Islam F Badgery-Parker T Foo F. The co-benefits of climate change mitigation strategies on cardiovascular health: a systematic review. Lancet Reg Health West Pac. (2024) 48:101098. doi: 10.1016/J.Lanwpc.2024.101098, PMID: 39380746 PMC11458989

[66] Landrigan PJ Fuller R Acosta N Acosta NJR Adeyi O Arnold R. The lancet commission on pollution and health. Lancet. (2018) 391:462–512. doi: 10.1016/S0140-6736(17)32345-0, PMID: 29056410

