# Supplementary material for: Burden of heart failure attributable to chronic kidney disease in older adults (1990–2021): an analysis from the global burden of disease study
Source: Front Public Health. 2025 Jun 18;13:1606719. doi: 10.3389/fpubh.2025.1606719 (PMC12213463; doi:10.3389/fpubh.2025.1606719)
Supplement: Supplementary file 1 [file Table_1.docx]

**Supplementary table S1** Prevalence of heart failure attributable to chronic kidney disease among older adults and corresponding AAPCs from 1990 to 2021 at the regional level.

| Region | Case,1990 | Prevalence,1990 | Case,2021 | Prevalence,2021 | AAPC, 1990-2021 | P value |
| --- | --- | --- | --- | --- | --- | --- |
| Andean Latin America | 3609.09 (2405.58-5226.87) | 163.45 (108.6-237.41) | 23613.76 (15232.97-35486.07) | 335.05 (216.04-503.68) | 2.45 (2.4 to 2.51) | <0.001 |
| Australasia | 1450.63 (887.04-2224.11) | 49.3 (30.07-75.8) | 9319.41 (5616.38-14316) | 121.22 (73.35-185.84) | 3.21 (3.06 to 3.34) | <0.001 |
| Caribbean | 2044.08 (1388.64-2897.6) | 67.43 (45.54-96.17) | 9536.89 (6126.22-14283.05) | 139.86 (90.21-208.74) | 2.42 (2.36 to 2.48) | <0.001 |
| Central Asia | 446.31 (274.03-673.74) | 8.61 (5.27-13.07) | 2154.94 (1244.4-3378.86) | 25 (14.2-39.54) | 3.53 (3.48 to 3.58) | <0.001 |
| Central Europe | 4731.83 (3049.19-6971.58) | 26.66 (17.07-39.44) | 12755.26 (7861.44-19419.58) | 41.04 (25.28-62.49) | 1.66 (1.47 to 1.85) | <0.001 |
| Central Latin America | 11404.36 (7552.29-16721.29) | 129.85 (85.54-190.75) | 80230.63 (50700-121691.41) | 266.72 (168.5-404.27) | 2.43 (2.34 to 2.52) | <0.001 |
| Central Sub-Saharan Africa | 3295.24 (1625.02-5841.11) | 201.09 (99.66-352.75) | 9733.98 (4864.5-17054.1) | 237.29 (118.84-412.64) | 0.55 (0.52 to 0.58) | <0.001 |
| East Asia | 39169.41 (23918.16-60444.78) | 44.26 (26.88-68.31) | 193430.23 (112169.76-313621) | 71.12 (41.24-115.28) | 1.58 (1.52 to 1.63) | <0.001 |
| Eastern Europe | 2119.88 (1265.38-3346.7) | 6.05 (3.6-9.58) | 7933.36 (4373.43-13248.82) | 16.79 (9.22-28.13) | 3.33 (3.1 to 3.57) | <0.001 |
| Eastern Sub-Saharan Africa | 11643.13 (5789.12-20560.01) | 181.29 (90.49-317.17) | 37241.31 (18801.5-64618.58) | 256.45 (129.84-442.04) | 1.14 (1.12 to 1.15) | <0.001 |
| High-income Asia Pacific | 10669.55 (5382.16-17328.46) | 48.05 (24.33-78.2) | 73320.83 (41623.67-116198.66) | 94.62 (54.89-148.49) | 2.23 (2.2 to 2.26) | <0.001 |
| High-income North America | 20206.52 (11307.15-32413.85) | 42.34 (23.73-67.88) | 139838.34 (78988.28-226950.82) | 153.07 (86.58-248.21) | 4.38 (4.31 to 4.47) | <0.001 |
| North Africa and Middle East | 12311.95 (8489.8-17270.2) | 78.24 (53.37-110.55) | 57070.7 (37673.56-84138.18) | 126.17 (82.59-186.76) | 1.56 (1.54 to 1.58) | <0.001 |
| Oceania | 86.53 (57.11-128.48) | 37.31 (24.32-55.65) | 364.77 (226.37-558.79) | 59.93 (36.74-92.42) | 1.54 (1.5 to 1.56) | <0.001 |
| South Asia | 19632.01 (12179.85-30204.46) | 39.25 (24.16-60.32) | 82377.49 (45390.58-135352.22) | 53.56 (29.39-87.88) | 1.02 (0.99 to 1.05) | <0.001 |
| Southeast Asia | 17086.03 (11546.14-24501.01) | 70.57 (47.38-101.53) | 95605.46 (63103.17-140470.76) | 142.91 (93.68-210.72) | 2.29 (2.26 to 2.32) | <0.001 |
| Southern Latin America | 3518.44 (2136.44-5354.21) | 64.69 (39.1-99.12) | 13112.12 (7253.33-21133.8) | 113.4 (62.89-182.57) | 1.87 (1.83 to 1.91) | <0.001 |
| Southern Sub-Saharan Africa | 4472.38 (2307.07-7600.38) | 165.34 (85.31-279.94) | 13131.88 (6769.95-22481.83) | 233.7 (120.48-396.9) | 1.11 (1.09 to 1.14) | <0.001 |
| Tropical Latin America | 5044.06 (3101.72-7775.45) | 55.27 (33.84-84.96) | 37476.88 (20772.54-61717.56) | 121.19 (67.29-199.13) | 2.59 (2.47 to 2.69) | <0.001 |
| Western Europe | 29042.47 (18404.09-42698.99) | 37.88 (24.03-55.89) | 141795.27 (86584.4-217332.4) | 95.33 (58.56-145.56) | 3.02 (2.89 to 3.14) | <0.001 |
| Western Sub-Saharan Africa | 32795.23 (18215.81-52995.82) | 403.34 (224.82-647.2) | 83794.43 (46605.52-134031.8) | 493.71 (275.73-783.84) | 0.68 (0.64 to 0.72) | <0.001 |

Abbreviations: AAPC, average annual percentage change
