# Supplementary material for: Burden of heart failure attributable to chronic kidney disease in older adults (1990–2021): an analysis from the global burden of disease study
Source: Front Public Health. 2025 Jun 18;13:1606719. doi: 10.3389/fpubh.2025.1606719 (PMC12213463; doi:10.3389/fpubh.2025.1606719)
Supplement: Supplementary file 2 [file Table_2.docx]

**Supplementary table S2** YLDs of heart failure attributable to chronic kidney disease among older adults and corresponding AAPCs from 1990 to 2021 at the regional level.

| Region | Number,1990 | YLDs,1990 | Number,2021 | YLDs,2021 | AAPC,1990-2021 | P value |
| --- | --- | --- | --- | --- | --- | --- |
| Andean Latin America | 449.3 (246.58-723.64) | 20.29 (11.13-32.77) | 2940.43 (1569.39-4884.49) | 41.7 (22.28-69.25) | 2.46 (2.4 to 2.52) | <0.001 |
| Australasia | 183.88 (94.26-312.37) | 6.23 (3.19-10.6) | 1169.46 (595.51-2062.47) | 15.24 (7.78-26.83) | 3.18 (3.04 to 3.31) | <0.001 |
| Caribbean | 258.05 (143.1-412.52) | 8.48 (4.72-13.6) | 1193.06 (651.73-2008.21) | 17.51 (9.59-29.38) | 2.41 (2.37 to 2.46) | <0.001 |
| Central Asia | 57.68 (29.73-96.16) | 1.11 (0.57-1.87) | 278.03 (137.17-485.86) | 3.22 (1.57-5.67) | 3.52 (3.47 to 3.57) | <0.001 |
| Central Europe | 607.62 (326.52-1002.06) | 3.41 (1.83-5.64) | 1627.36 (852.68-2810.55) | 5.24 (2.74-9.05) | 1.63 (1.44 to 1.8) | <0.001 |
| Central Latin America | 1426.26 (806.19-2306.36) | 16.18 (9.14-26.15) | 10021.42 (5440.45-16792.94) | 33.29 (18.06-55.75) | 2.43 (2.35 to 2.51) | <0.001 |
| Central Sub-Saharan Africa | 411.14 (174.86-823.12) | 24.75 (10.78-48.51) | 1214.36 (521.56-2385.85) | 29.33 (12.84-57.02) | 0.54 (0.52 to 0.56) | <0.001 |
| East Asia | 5032.67 (2683.71-8712.14) | 5.65 (3.01-9.77) | 24534.87 (12665.32-43710.05) | 9 (4.64-16.01) | 1.55 (1.5 to 1.6) | <0.001 |
| Eastern Europe | 274.29 (142.83-474.86) | 0.78 (0.41-1.36) | 1024.46 (489.19-1879.04) | 2.17 (1.03-3.99) | 3.33 (3.1 to 3.55) | <0.001 |
| Eastern Sub-Saharan Africa | 1442.87 (640.88-2842.92) | 22.2 (9.98-43.35) | 4613.05 (2096.27-8908.95) | 31.5 (14.44-60.23) | 1.14 (1.12 to 1.15) | <0.001 |
| High-income Asia Pacific | 1360.13 (611.87-2442.21) | 6.09 (2.77-10.96) | 9187.21 (4500.16-16373.73) | 11.94 (5.9-21.13) | 2.21 (2.18 to 2.24) | <0.001 |
| High-income North America | 2587.25 (1259.62-4610.96) | 5.42 (2.65-9.65) | 17573.64 (8750.32-32078.47) | 19.25 (9.59-35.1) | 4.28 (4.21 to 4.34) | <0.001 |
| North Africa and Middle East | 1547.71 (876.92-2435.35) | 9.75 (5.54-15.42) | 7131.85 (3905.49-11673.56) | 15.68 (8.6-25.74) | 1.55 (1.53 to 1.57) | <0.001 |
| Oceania | 11.06 (6.04-18.47) | 4.71 (2.58-7.85) | 46.23 (24.42-79.61) | 7.52 (3.96-12.97) | 1.52 (1.49 to 1.55) | <0.001 |
| South Asia | 2495.43 (1334.86-4277.81) | 4.92 (2.64-8.42) | 10407.66 (5161.05-18925.76) | 6.71 (3.32-12.18) | 1.02 (0.99 to 1.05) | <0.001 |
| Southeast Asia | 2149.42 (1213.9-3490) | 8.8 (4.98-14.31) | 11923.47 (6592.19-19792.61) | 17.72 (9.8-29.46) | 2.28 (2.24 to 2.31) | <0.001 |
| Southern Latin America | 447.02 (217.89-761.57) | 8.19 (4.01-14.01) | 1644.81 (791.62-2919.17) | 14.23 (6.86-25.24) | 1.84 (1.81 to 1.88) | <0.001 |
| Southern Sub-Saharan Africa | 557.56 (256.12-1051.16) | 20.51 (9.46-38.49) | 1640.29 (756.25-3098.44) | 29.02 (13.46-54.46) | 1.12 (1.09 to 1.14) | <0.001 |
| Tropical Latin America | 640.35 (335.5-1106.44) | 6.96 (3.65-12.01) | 4673.19 (2306.26-8483.2) | 15.09 (7.46-27.36) | 2.54 (2.41 to 2.65) | <0.001 |
| Western Europe | 3688.47 (2008.43-6011.73) | 4.8 (2.62-7.84) | 17750.25 (9641.85-30197.92) | 11.98 (6.51-20.36) | 2.99 (2.87 to 3.1) | <0.001 |
| Western Sub-Saharan Africa | 4034.89 (1993.64-7353.98) | 49.26 (24.54-89.01) | 10354.64 (5161.42-18770.74) | 60.64 (30.47-109.11) | 0.7 (0.66 to 0.73) | <0.001 |

Abbreviations: YLDs, years lived with disability; AAPC, average annual percentage change
