# Supplementary material for: Burden of heart failure attributable to chronic kidney disease in older adults (1990–2021): an analysis from the global burden of disease study
Source: Front Public Health. 2025 Jun 18;13:1606719. doi: 10.3389/fpubh.2025.1606719 (PMC12213463; doi:10.3389/fpubh.2025.1606719)
Supplement: Supplementary file 3 [file Table_3.docx]

**Supplementary table S3** Prevalence of heart failure attributable to chronic kidney disease among older adults and corresponding AAPCs from 1990 to 2021 at the country/territory level.

| Country and territory | Case,1990 | Prevalence,1990 | Case,2021 | Prevalence,2021 | AAPC,1990-2021 | P value |
| --- | --- | --- | --- | --- | --- | --- |
| Afghanistan | 365.15 (242.09-526.07) | 54.3 (35.63-79.09) | 569.73 (351.45-873.72) | 73.26 (45.18-112.55) | 0.98 (0.94 to 1.01) | <0.001 |
| Albania | 74.74 (46.68-110.12) | 33.61 (20.83-49.76) | 245.18 (148.78-382.01) | 42.61 (25.7-66.72) | 0.78 (0.75 to 0.81) | <0.001 |
| Algeria | 630.13 (418.95-900.91) | 52.65 (34.81-76.26) | 3676.08 (2329.78-5536.87) | 100.05 (62.64-152.26) | 2.13 (2.09 to 2.17) | <0.001 |
| American Samoa | 1.95 (1.26-2.87) | 99.8 (64.02-147.53) | 11.34 (6.87-17.35) | 235.97 (141.64-363.16) | 2.89 (2.82 to 2.97) | <0.001 |
| Andorra | 3.58 (2.21-5.37) | 58.56 (35.63-88.79) | 17.4 (9.95-27.65) | 81.21 (46.93-127.87) | 1.06 (1.02 to 1.09) | <0.001 |
| Angola | 436.79 (212.78-778.14) | 162.94 (79.4-288.46) | 1961.68 (953.92-3438.43) | 229.54 (112.53-398.5) | 1.12 (1.11 to 1.14) | <0.001 |
| Antigua and Barbuda | 7.92 (5.4-11.02) | 107.15 (73.11-149.31) | 20.83 (13.11-31.57) | 171.81 (107.57-261.22) | 1.61 (1.41 to 1.79) | <0.001 |
| Argentina | 2661.85 (1617.25-4072.76) | 69.58 (42.05-107.18) | 7986.39 (4374.69-12968.99) | 107.68 (59.15-174.62) | 1.33 (1.25 to 1.39) | <0.001 |
| Armenia | 5.55 (3.43-8.46) | 1.92 (1.17-2.95) | 215.24 (138.19-320.86) | 38 (24.22-56.77) | 10.48 (10.19 to 10.8) | <0.001 |
| Australia | 1291.59 (786.92-1975.05) | 52.85 (32.13-81.04) | 8385.95 (5043.74-12804.42) | 128.48 (77.65-195.68) | 3.2 (3.02 to 3.35) | <0.001 |
| Austria | 498.31 (318.61-710.12) | 31.52 (20.21-44.85) | 2569.73 (1480.26-3943.66) | 89.88 (52.18-137.39) | 3.49 (3.29 to 3.66) | <0.001 |
| Azerbaijan | 46.95 (28.02-72.26) | 9.04 (5.36-13.99) | 216.11 (119.7-356.42) | 20.65 (11.22-34.53) | 2.75 (2.68 to 2.8) | <0.001 |
| Bahamas | 9.1 (5.88-13.41) | 54.57 (35.26-80.62) | 43.68 (26.22-69.41) | 100.32 (60.02-159.4) | 2.04 (1.85 to 2.19) | <0.001 |
| Bahrain | 9.16 (6.32-13.01) | 68.59 (46.64-98.36) | 122.42 (83-177.2) | 172.92 (114.83-254.61) | 3.03 (2.99 to 3.07) | <0.001 |
| Bangladesh | 1371.84 (914.7-1985.2) | 29.9 (19.83-43.62) | 6216.85 (3791.92-9538.52) | 42.04 (25.58-64.96) | 1.07 (1.03 to 1.1) | <0.001 |
| Barbados | 39.25 (26.5-56.83) | 94.21 (63.64-136.6) | 128.91 (82.6-193.18) | 188.85 (120.84-283.36) | 2.36 (2.27 to 2.46) | <0.001 |
| Belarus | 23.31 (14.66-34.64) | 1.47 (0.92-2.19) | 142.98 (86.04-226.83) | 6.64 (3.98-10.57) | 5.34 (5.11 to 5.55) | <0.001 |
| Belgium | 664.21 (397.95-1003.22) | 32.71 (19.63-49.61) | 2166.57 (1358.5-3302.69) | 57.48 (36.23-86.87) | 1.75 (1.49 to 1.97) | <0.001 |
| Belize | 13.09 (8.62-19.05) | 115.69 (76.36-168.05) | 65.35 (40.47-100.77) | 208.71 (129.08-321.9) | 2.08 (1.99 to 2.16) | <0.001 |
| Benin | 388.9 (194.76-683.93) | 188.19 (94.09-329.44) | 1166.55 (593.84-2009.85) | 248.78 (126.89-426.85) | 0.9 (0.88 to 0.92) | <0.001 |
| Bermuda | 4.39 (2.86-6.4) | 59.07 (38.37-86.42) | 29 (18.06-43.75) | 152.73 (95.31-229.72) | 3.1 (3.04 to 3.16) | <0.001 |
| Bhutan | 8.43 (5.76-11.97) | 41.16 (27.7-59.03) | 47.69 (31.34-70.17) | 70.39 (46.03-103.96) | 1.74 (1.71 to 1.76) | <0.001 |
| Bolivia (Plurinational State of) | 444.81 (294.02-645.1) | 136.83 (90.01-199.65) | 2844.33 (1815.49-4280.17) | 284.72 (181.09-429.93) | 2.4 (2.36 to 2.43) | <0.001 |
| Bosnia and Herzegovina | 104.55 (62.49-160.29) | 25.14 (14.88-38.98) | 323.1 (177.71-513.42) | 38.5 (21.04-61.36) | 1.41 (1.37 to 1.44) | <0.001 |
| Botswana | 58.51 (28.54-105.22) | 127.17 (62.21-225.98) | 272.63 (131.51-478.8) | 215.3 (104.27-376.36) | 1.72 (1.69 to 1.73) | <0.001 |
| Brazil | 4867.4 (2963.35-7541.95) | 54.81 (33.22-84.73) | 36620.59 (20181.48-60570.29) | 121.04 (66.82-199.75) | 2.61 (2.49 to 2.71) | <0.001 |
| Brunei Darussalam | 3.92 (1.36-7.08) | 46.01 (16.8-83.13) | 19.07 (6.21-35.39) | 70.13 (23.81-130.41) | 1.35 (1.32 to 1.36) | <0.001 |
| Bulgaria | 146.39 (91.96-214.44) | 9.89 (6.12-14.75) | 434.24 (244.01-716.2) | 21.37 (12.01-35.35) | 2.65 (2.51 to 2.76) | <0.001 |
| Burkina Faso | 935.87 (467.1-1645.09) | 240.2 (120.43-418.28) | 2348.47 (1197.92-4051.07) | 274.79 (140.49-471.8) | 0.45 (0.43 to 0.48) | <0.001 |
| Burundi | 303.01 (146.53-546.98) | 138.92 (67.18-249.65) | 814.94 (394.89-1465.67) | 205.77 (99.96-368.33) | 1.29 (1.28 to 1.31) | <0.001 |
| Cabo Verde | 50.1 (25.31-86.67) | 163.6 (82.34-284.08) | 138.18 (70.8-238) | 284.63 (145.66-489.85) | 1.81 (1.78 to 1.84) | <0.001 |
| Cambodia | 159.26 (105.02-230.82) | 39.4 (25.79-57.65) | 726.73 (459.39-1086.16) | 60.81 (38.16-91.48) | 1.41 (1.38 to 1.44) | <0.001 |
| Cameroon | 1038.71 (524.17-1801.96) | 272.76 (137.5-471.82) | 3502.48 (1807.95-5935.32) | 333.29 (173.02-561.7) | 0.65 (0.6 to 0.7) | <0.001 |
| Canada | 1870.39 (1178.51-2806.98) | 44.64 (28.05-67.1) | 14865.64 (8565.26-23661.47) | 143.46 (82.96-227.49) | 3.77 (3.65 to 3.89) | <0.001 |
| Central African Republic | 125.03 (61.19-222.13) | 162.12 (79.46-284.15) | 253.69 (125.73-447.29) | 185.22 (92.12-324.51) | 0.44 (0.43 to 0.45) | <0.001 |
| Chad | 459.32 (230.05-814.56) | 157.93 (78.97-278.24) | 897.85 (454.42-1546.22) | 182.17 (92.16-312.93) | 0.46 (0.45 to 0.47) | <0.001 |
| Chile | 623.41 (366.13-970.63) | 56.31 (32.88-88.35) | 4482.55 (2495.69-7256.33) | 134.48 (74.88-217.72) | 2.95 (2.84 to 3.04) | <0.001 |
| China | 36574.25 (21775.18-57201.07) | 42.6 (25.23-66.74) | 176495.45 (98547.04-292023.73) | 66.66 (37.18-110.41) | 1.5 (1.44 to 1.57) | <0.001 |
| Colombia | 1116.84 (746.97-1625.37) | 62.08 (41.29-90.63) | 8349.8 (5415.18-12703.71) | 119.16 (77.64-180.51) | 2.15 (2.09 to 2.21) | <0.001 |
| Comoros | 31.3 (15.1-56.8) | 184.04 (88.98-330.85) | 127.97 (62.54-226.75) | 269.73 (132.05-476.74) | 1.26 (1.25 to 1.27) | <0.001 |
| Congo | 175.89 (88.17-312.81) | 208.85 (104.34-365.98) | 556.31 (279.11-971.08) | 276.42 (138.15-480.23) | 0.92 (0.9 to 0.93) | <0.001 |
| Cook Islands | 0.36 (0.22-0.55) | 29.57 (18.22-45.25) | 2.15 (1.27-3.48) | 66.61 (38.98-108.07) | 2.64 (2.58 to 2.68) | <0.001 |
| Costa Rica | 149.15 (99.54-215.92) | 73.89 (49.25-107.09) | 1661.87 (1046.92-2496.35) | 240.79 (152.14-360.82) | 3.98 (3.92 to 4.05) | <0.001 |
| Coted'Ivoire | 615.29 (311.23-1086.86) | 218.06 (110.1-383.87) | 2326.13 (1197.94-3993.7) | 262.84 (136.02-448.71) | 0.61 (0.59 to 0.63) | <0.001 |
| Croatia | 119.08 (68.44-183.16) | 16.94 (9.55-26.4) | 531.4 (289.22-848.37) | 41.51 (22.58-66.33) | 2.78 (2.59 to 2.94) | <0.001 |
| Cuba | 440.45 (297.34-625.18) | 34.48 (23.19-49.18) | 2399.76 (1528.89-3635.48) | 93.46 (59.8-141.13) | 3.31 (3.22 to 3.39) | <0.001 |
| Cyprus | 38.01 (20.67-59.27) | 46.11 (24.78-73.27) | 193.25 (109.47-308.51) | 73.92 (41.54-118.95) | 1.8 (1.41 to 2.18) | <0.001 |
| Czechia | 430.89 (262.45-640.55) | 24.25 (14.79-36.25) | 1479.73 (831.08-2403.71) | 48.06 (27.14-77.84) | 2.3 (1.9 to 2.65) | <0.001 |
| Democratic People's Republic of Korea | 566.9 (384.61-802.94) | 36.35 (24.45-51.97) | 1716.48 (1136-2554.32) | 45.4 (30-67.79) | 0.73 (0.72 to 0.74) | <0.001 |
| Democratic Republic of the Congo | 2402.2 (1181.32-4306.44) | 211.82 (104.5-374.62) | 6498.15 (3234.43-11427.82) | 233.64 (116.88-407.38) | 0.33 (0.29 to 0.37) | <0.001 |
| Denmark | 144.68 (84.64-223.02) | 12.99 (7.64-20) | 1499.31 (829.85-2433.1) | 85.13 (47.5-137.35) | 6.49 (6.32 to 6.64) | <0.001 |
| Djibouti | 14.57 (6.95-26.36) | 156.05 (74.83-279.4) | 120.04 (58.82-215.6) | 253.27 (123.78-450.85) | 1.59 (1.56 to 1.61) | <0.001 |
| Dominica | 5.75 (3.75-8.36) | 74.48 (48.53-108.57) | 13.36 (8.15-20.68) | 133.85 (81.46-207.65) | 1.96 (1.92 to 1.99) | <0.001 |
| Dominican Republic | 256.76 (169.17-370.16) | 66.33 (43.46-96.1) | 1297.11 (779.73-2044.59) | 108.9 (65.38-171.78) | 1.56 (1.43 to 1.63) | <0.001 |
| Ecuador | 647.15 (422.03-953.7) | 111.71 (72.55-165.41) | 5480.09 (3404.73-8476) | 276.56 (171.43-428.32) | 3.01 (2.94 to 3.08) | <0.001 |
| Egypt | 1567.93 (1057.13-2237.41) | 71.15 (47.33-102.72) | 6545.23 (4180.22-9884.77) | 125.17 (78.35-191.25) | 1.86 (1.78 to 1.93) | <0.001 |
| El Salvador | 463.36 (315.36-666.75) | 133.09 (90.62-191.28) | 3410.51 (2217.54-5043.87) | 426.23 (278.59-627.45) | 3.92 (3.81 to 4.03) | <0.001 |
| Equatorial Guinea | 26.06 (12.85-45.71) | 173.94 (85.78-302.67) | 142.89 (71.22-249.01) | 350.72 (175.46-608.63) | 2.33 (2.28 to 2.38) | <0.001 |
| Eritrea | 79.08 (38.29-142.34) | 123.04 (59.52-219.15) | 377.45 (184.56-677.17) | 188.63 (92.08-337.25) | 1.46 (1.42 to 1.51) | <0.001 |
| Estonia | 27.22 (16.88-41.29) | 10.54 (6.52-16.06) | 246.48 (122.76-433.92) | 61.45 (30.75-107.96) | 5.17 (4.85 to 5.45) | <0.001 |
| Eswatini | 42.19 (20.81-75.73) | 176.59 (86.61-314.2) | 110.09 (54.9-195.49) | 243.59 (122.02-428.57) | 1.03 (1 to 1.05) | <0.001 |
| Ethiopia | 3591.79 (1760.23-6408.72) | 233.82 (115.82-410.51) | 13221.38 (6615.5-23239.76) | 323.74 (162.46-565.92) | 1.07 (1.05 to 1.08) | <0.001 |
| Fiji | 14.83 (9.7-21.89) | 50.22 (32.47-74.61) | 74.33 (46.98-112.39) | 103.98 (64.65-158.82) | 2.35 (2.33 to 2.37) | <0.001 |
| Finland | 89.41 (49.19-137.92) | 9.86 (5.43-15.26) | 645.84 (353.96-1085.28) | 31.47 (17.45-52.37) | 3.92 (3.63 to 4.21) | <0.001 |
| France | 9279.44 (5995.97-13566.03) | 81.23 (52.45-119.06) | 43383.71 (26119.44-67304.3) | 186.19 (113.38-286.14) | 2.64 (2.42 to 2.86) | <0.001 |
| Gabon | 129.26 (64.75-224.41) | 226.68 (113.25-391.03) | 321.25 (160.43-561.23) | 357.03 (178.49-620.86) | 1.48 (1.46 to 1.49) | <0.001 |
| Gambia | 59.45 (30.17-104.3) | 196.41 (99.59-341.89) | 228.98 (117.87-391.59) | 247.36 (127.27-422.76) | 0.72 (0.71 to 0.74) | <0.001 |
| Georgia | 33.76 (19.69-51.79) | 4.42 (2.57-6.8) | 161.44 (61.1-286.82) | 19.84 (7.45-35.21) | 4.95 (4.66 to 5.23) | <0.001 |
| Germany | 4586.7 (2824.02-6917.6) | 27.18 (16.79-41.02) | 29700.66 (16526.88-48209.94) | 95.03 (53.02-154.31) | 4.29 (4.04 to 4.53) | <0.001 |
| Ghana | 473.8 (230.97-851.64) | 92.98 (45.16-166.64) | 2258.63 (1072.59-4100.93) | 157.65 (75.11-285.5) | 1.75 (1.72 to 1.77) | <0.001 |
| Greece | 1223.84 (756.18-1826.48) | 65.94 (40.51-99.18) | 2435.69 (1354.19-3879.75) | 62.36 (34.95-99.51) | -0.01 (-0.23 to 0.19) | 0.875 |
| Greenland | 0.84 (0.39-1.41) | 32.9 (14.93-56) | 4.46 (1.99-7.91) | 68.75 (29.52-123.38) | 2.5 (2.39 to 2.59) | <0.001 |
| Grenada | 10.15 (6.6-14.83) | 99.21 (64.68-144.8) | 23.49 (14.38-35.72) | 183.52 (112.34-279.2) | 2.26 (2.15 to 2.38) | <0.001 |
| Guam | 3.66 (2.34-5.56) | 57.92 (36.6-88.96) | 36.39 (20.96-59.47) | 130.01 (75.81-210.31) | 2.65 (2.6 to 2.69) | <0.001 |
| Guatemala | 548.36 (357.61-819.53) | 161.73 (104.56-242.96) | 4695.25 (3013.38-7009.52) | 362.08 (231.5-542.39) | 2.62 (2.56 to 2.69) | <0.001 |
| Guinea | 630.31 (315.87-1096.28) | 187.36 (93.94-323.96) | 1141.11 (578.68-1963.39) | 211.32 (107.21-362.56) | 0.38 (0.36 to 0.4) | <0.001 |
| Guinea-Bissau | 59.11 (29.58-105.14) | 184.91 (93.14-324.75) | 109.75 (55.76-190.95) | 208.55 (106.42-359.34) | 0.37 (0.33 to 0.41) | <0.001 |
| Guyana | 16.73 (10.95-24.58) | 44.63 (28.98-66.11) | 72.02 (45.09-111.31) | 108.74 (67.61-168.66) | 3.15 (3 to 3.33) | <0.001 |
| Haiti | 144.61 (96.41-210.14) | 48.23 (31.89-70.48) | 452.26 (290.48-679.74) | 69.72 (44.36-105.49) | 1.2 (1.19 to 1.21) | <0.001 |
| Honduras | 105.99 (72.19-149.85) | 48.81 (33.06-69.36) | 539.93 (345.22-806.52) | 77.61 (49.38-116.28) | 1.52 (1.47 to 1.56) | <0.001 |
| Hungary | 184.11 (100.25-288.16) | 9.94 (5.41-15.67) | 864.62 (459.1-1443.27) | 30.55 (16.26-50.91) | 3.67 (3.57 to 3.76) | <0.001 |
| Iceland | 2.66 (1.32-4.29) | 6.83 (3.41-10.97) | 19.73 (10.27-33.19) | 23.55 (12.42-39.25) | 3.5 (2.41 to 4.73) | <0.001 |
| India | 13422.24 (8021.65-21024.96) | 34.18 (20.22-53.6) | 62723.35 (33186.4-105536.52) | 49.97 (26.32-84.13) | 1.25 (1.22 to 1.29) | <0.001 |
| Indonesia | 3881.8 (2326.61-6075.38) | 43.57 (26.04-68.07) | 13184.83 (7388.2-21853.74) | 57.19 (31.99-94.31) | 0.93 (0.85 to 1.01) | <0.001 |
| Iran (Islamic Republic of) | 1253.13 (738.93-1961.16) | 53.25 (31.14-83.43) | 7695.81 (4136.26-12884.54) | 92.98 (49.82-155.87) | 1.94 (1.88 to 2.01) | <0.001 |
| Iraq | 1129.15 (760.69-1604.8) | 130 (87.5-184.82) | 3435.82 (2270.56-5032.8) | 157.29 (103.25-231.74) | 0.63 (0.6 to 0.66) | <0.001 |
| Ireland | 105.06 (61.73-164.25) | 21.1 (12.39-33.14) | 713.59 (412.93-1140.08) | 65.59 (38.03-104.69) | 3.84 (3.71 to 3.94) | <0.001 |
| Israel | 373.05 (214.04-582.72) | 64.77 (37.22-101.76) | 3314.2 (1937.41-5155.25) | 188.17 (110.36-291.81) | 3.81 (3.63 to 4.01) | <0.001 |
| Italy | 3785.56 (2043.92-6138.46) | 32.92 (17.83-53.52) | 16975.52 (9355.26-28433.79) | 73.79 (40.88-123.11) | 2.69 (2.54 to 2.85) | <0.001 |
| Jamaica | 243.59 (156.93-358.04) | 102.05 (65.87-149.88) | 543.63 (339.87-824.75) | 134.18 (84.67-201.36) | 0.99 (0.9 to 1.07) | <0.001 |
| Japan | 9220.86 (4544.26-15239.2) | 47.47 (23.57-78.46) | 57217.4 (30613.99-93801.28) | 85.43 (46.33-139.59) | 1.95 (1.9 to 1.99) | <0.001 |
| Jordan | 90.25 (60.96-127.89) | 79.79 (53.35-114.37) | 942.57 (612.59-1399.94) | 142.73 (91.89-213.68) | 1.91 (1.87 to 1.95) | <0.001 |
| Kazakhstan | 115.76 (71.93-172.74) | 7.98 (4.94-11.94) | 562.66 (328.14-882.61) | 28.78 (16.58-45.53) | 4.34 (4.1 to 4.54) | <0.001 |
| Kenya | 1157.85 (574.91-2059.84) | 149.77 (74.57-265.3) | 3992.12 (1996.86-7072.76) | 198.41 (99.61-347.89) | 0.99 (0.91 to 1.07) | <0.001 |
| Kiribati | 2.3 (1.43-3.47) | 71.34 (43.98-108.47) | 6.54 (3.91-10.18) | 111.16 (65.68-173.86) | 1.49 (1.43 to 1.54) | <0.001 |
| Kuwait | 50.34 (32.81-74.15) | 108.03 (69.66-159.77) | 283.23 (182.23-428.95) | 115.53 (73.92-175.23) | 0.27 (0.16 to 0.38) | <0.001 |
| Kyrgyzstan | 19.16 (11.23-29.83) | 5.6 (3.26-8.77) | 115.58 (72.3-172.74) | 22.83 (14.07-34.5) | 4.69 (4.61 to 4.79) | <0.001 |
| Lao People's Democratic Republic | 109.68 (70.95-161.61) | 58.08 (37.32-86.21) | 387.35 (242.03-586.44) | 88.76 (54.99-135.34) | 1.37 (1.35 to 1.39) | <0.001 |
| Latvia | 23.28 (14.31-35.19) | 5.17 (3.17-7.84) | 144.78 (79.71-237.23) | 25.14 (13.93-41.08) | 5.88 (5.6 to 6.15) | <0.001 |
| Lebanon | 216.09 (147.24-308.53) | 98.73 (66.65-142.26) | 1867.25 (1242.01-2700.55) | 232.83 (155.2-336) | 2.91 (2.85 to 2.97) | <0.001 |
| Lesotho | 103.59 (51.43-183.66) | 120.83 (59.98-213.24) | 177.03 (87.87-312.3) | 185.32 (92.17-323.64) | 1.4 (1.38 to 1.41) | <0.001 |
| Liberia | 268.38 (136.4-477.81) | 236.1 (119.92-415.88) | 498.82 (256.8-859.35) | 289.81 (149.35-498.09) | 0.66 (0.62 to 0.7) | <0.001 |
| Libya | 205.66 (140.37-296.06) | 104.13 (70.9-149.95) | 740.31 (466.29-1116.98) | 150.88 (94.75-227.65) | 1.18 (1.1 to 1.23) | <0.001 |
| Lithuania | 25.65 (15.8-38.96) | 4.55 (2.79-6.96) | 189.45 (102.84-309.75) | 23.64 (12.92-38.71) | 5.1 (4.79 to 5.33) | <0.001 |
| Luxembourg | 21.96 (11.61-35.13) | 33.15 (17.63-53.17) | 157.86 (87.47-256.02) | 105.16 (58.67-169.68) | 3.72 (3.49 to 3.9) | <0.001 |
| Madagascar | 484.61 (232.5-876.84) | 106.24 (51.03-191.32) | 1119.76 (545.52-2018.28) | 136.78 (66.68-244.24) | 0.83 (0.82 to 0.85) | <0.001 |
| Malawi | 577.21 (276.39-1041.07) | 176.83 (85.13-316.07) | 1530.51 (737.41-2717.24) | 235.62 (113.86-416.35) | 0.94 (0.93 to 0.95) | <0.001 |
| Malaysia | 683.41 (454.41-984) | 71.17 (47.25-102.62) | 4211.64 (2768.48-6206.59) | 135.83 (88.88-200.95) | 2.08 (2.04 to 2.11) | <0.001 |
| Maldives | 8.77 (5.88-12.75) | 124.75 (82.09-184.52) | 60.01 (38.53-89.68) | 188.76 (120.54-283.46) | 1.31 (1.28 to 1.34) | <0.001 |
| Mali | 720.71 (358.44-1277.57) | 221.46 (110.75-387.57) | 1959.75 (978.78-3426.79) | 263.9 (132.63-458.01) | 0.56 (0.55 to 0.57) | <0.001 |
| Malta | 16.76 (10.41-25.34) | 34.72 (21.38-52.86) | 123.77 (70.52-199.84) | 84.49 (48.42-135.82) | 2.8 (2.6 to 2.95) | <0.001 |
| Marshall Islands | 0.83 (0.53-1.23) | 54.55 (34.53-81.57) | 2.76 (1.69-4.26) | 103.96 (62.92-160.98) | 2.15 (2.11 to 2.19) | <0.001 |
| Mauritania | 223.07 (111.72-387.8) | 223.59 (112.54-387.29) | 611.57 (308.42-1062.55) | 284.38 (143.34-493.15) | 0.76 (0.73 to 0.79) | <0.001 |
| Mauritius | 74.09 (46.32-111.53) | 99.97 (62.14-151.32) | 680.24 (443.09-990.09) | 297.31 (193.06-434.62) | 3.61 (3.54 to 3.67) | <0.001 |
| Mexico | 8148.52 (5128.5-12431.25) | 183.95 (114.95-281.48) | 52553.08 (30939.67-83450.93) | 351.38 (206.26-558.65) | 2.16 (2.08 to 2.24) | <0.001 |
| Micronesia (Federated States of) | 2.58 (1.63-3.89) | 52.48 (32.96-79.45) | 6.66 (4.01-10.46) | 107.93 (64.21-170.15) | 2.38 (2.33 to 2.43) | <0.001 |
| Monaco | 2.83 (1.77-4.26) | 26.62 (16.71-40.26) | 10.45 (6.04-16.55) | 67.9 (39.72-106.24) | 3.12 (2.99 to 3.22) | <0.001 |
| Mongolia | 27.93 (15.18-43.67) | 23.72 (12.86-37.23) | 73.62 (39.3-120.21) | 35.28 (18.57-58.25) | 1.26 (1.18 to 1.33) | <0.001 |
| Montenegro | 29.87 (18.49-45.12) | 41.68 (25.76-63.05) | 64.45 (38.69-99.11) | 51.31 (30.46-79.52) | 0.68 (0.64 to 0.71) | <0.001 |
| Morocco | 1902.06 (1267.17-2741.41) | 127.13 (84.18-184.31) | 6534.2 (4109.38-10038.62) | 180.59 (112.74-278.69) | 1.12 (1.1 to 1.15) | <0.001 |
| Mozambique | 852.21 (439.45-1488.76) | 181.21 (93.19-313.1) | 2131.73 (1091.8-3631.46) | 249.77 (129.24-420.85) | 1.05 (1.03 to 1.06) | <0.001 |
| Myanmar | 808.47 (530.76-1188.73) | 37.89 (24.67-56.21) | 2992.86 (1859.6-4638.24) | 59.84 (36.94-92.95) | 1.49 (1.44 to 1.53) | <0.001 |
| Namibia | 65.77 (31.99-117.91) | 119.39 (58.35-211.66) | 208.28 (102.17-362.83) | 169.48 (83.73-294.54) | 1.14 (1.11 to 1.16) | <0.001 |
| Nauru | 0.18 (0.11-0.27) | 52.15 (32.46-78.66) | 0.4 (0.23-0.64) | 82.69 (48.13-131.81) | 1.46 (1.38 to 1.51) | <0.001 |
| Nepal | 418.9 (284.01-603.32) | 51.52 (34.44-74.86) | 2057.65 (1298.18-3132.18) | 86.28 (54.19-131.79) | 1.66 (1.62 to 1.69) | <0.001 |
| Netherlands | 720.46 (464.22-1057.56) | 27.5 (17.7-40.48) | 4559.17 (2611.2-7287.04) | 89.13 (51.13-142.24) | 4.06 (3.8 to 4.29) | <0.001 |
| New Zealand | 159.04 (87.71-258.97) | 31.97 (17.61-52.21) | 933.45 (508.4-1573.22) | 80.35 (43.84-135.32) | 3.13 (2.97 to 3.28) | <0.001 |
| Nicaragua | 246.07 (165.11-351.6) | 149.56 (100.17-213.98) | 1923.97 (1252.38-2852.3) | 345.85 (224.29-514.17) | 2.78 (2.73 to 2.82) | <0.001 |
| Niger | 368.63 (184.23-654.13) | 170.74 (85.53-299.7) | 1285.79 (640.38-2227.38) | 191.09 (95.97-330.25) | 0.37 (0.35 to 0.39) | <0.001 |
| Nigeria | 25157.47 (14287.3-39824.18) | 589.66 (335.63-925.75) | 61636.82 (35039.8-95834.05) | 775.85 (442.28-1196.81) | 0.85 (0.78 to 0.9) | <0.001 |
| Niue | 0.2 (0.12-0.3) | 65.24 (41.32-98.37) | 0.35 (0.21-0.55) | 137.11 (81.91-214.86) | 2.42 (2.39 to 2.45) | <0.001 |
| North Macedonia | 38.81 (23.75-57.93) | 18.61 (11.33-27.88) | 117.16 (66.53-186.47) | 28.36 (15.91-45.41) | 1.4 (1.37 to 1.44) | <0.001 |
| Northern Mariana Islands | 1.75 (1.15-2.54) | 160.28 (103.77-234.55) | 12.1 (7.4-18.87) | 258.04 (154.42-404.21) | 1.53 (1.48 to 1.57) | <0.001 |
| Norway | 119.85 (49.05-212.36) | 12.3 (5.14-21.71) | 785.38 (367.68-1381.8) | 52.65 (24.87-92.16) | 5.01 (4.87 to 5.18) | <0.001 |
| Oman | 26.05 (18.2-36.12) | 42.58 (29.51-59.38) | 170.37 (112.41-250.55) | 110.25 (72.26-163.29) | 3.14 (3.08 to 3.19) | <0.001 |
| Pakistan | 4410.6 (2644.75-6908.42) | 77.7 (46.36-121.51) | 11331.94 (6266.46-18891.82) | 103.99 (57.28-173.1) | 0.92 (0.9 to 0.94) | <0.001 |
| Palau | 1.07 (0.68-1.6) | 107.85 (68.76-162.44) | 3.98 (2.46-6.1) | 196.56 (120.19-303.73) | 1.98 (1.95 to 2.02) | <0.001 |
| Palestine | 77.07 (52.7-108.25) | 83.99 (57.02-119.04) | 293.04 (193.9-425.75) | 124.2 (81.8-182) | 1.26 (1.23 to 1.28) | <0.001 |
| Panama | 146.97 (99.52-212.48) | 85.45 (57.92-123.5) | 1534.57 (990.13-2278.7) | 275.41 (178.33-407.5) | 3.98 (3.92 to 4.05) | <0.001 |
| Papua New Guinea | 35.76 (23.38-53.36) | 25.17 (16.21-37.78) | 141.28 (87.07-216.95) | 35.49 (21.67-54.63) | 1.12 (1.08 to 1.14) | <0.001 |
| Paraguay | 176.66 (116.98-254.01) | 71.19 (47.14-102.4) | 856.29 (527.65-1307.01) | 127.96 (78.98-195.05) | 1.98 (1.92 to 2.04) | <0.001 |
| Peru | 2517.13 (1662.79-3680.45) | 191.64 (126.38-280.58) | 15289.33 (9826.29-22918.67) | 374.48 (241.17-560.04) | 2.22 (2.13 to 2.3) | <0.001 |
| Philippines | 2072.04 (1231.12-3259.6) | 77.6 (45.89-121.78) | 9171.79 (4947.68-15442.84) | 110.73 (59.68-186.14) | 1.16 (1.14 to 1.18) | <0.001 |
| Poland | 2587.95 (1561.13-4002.56) | 48.6 (29.26-75.35) | 6045.09 (3441.82-9672.36) | 60.44 (34.4-96.87) | 1.33 (0.95 to 1.67) | <0.001 |
| Portugal | 855.03 (547.73-1254.56) | 50.68 (32.44-74.8) | 4755.47 (2820.15-7406.59) | 120.44 (71.72-187.17) | 3.1 (2.99 to 3.2) | <0.001 |
| Puerto Rico | 675.26 (450.01-983.59) | 148.13 (98.41-216.52) | 3418.72 (2135.5-5164.39) | 313.52 (198.34-468.83) | 2.43 (2.35 to 2.52) | <0.001 |
| Qatar | 4.35 (2.98-6.12) | 67.02 (45.04-95.63) | 108.73 (72.53-157.4) | 201.06 (131.38-295.1) | 3.6 (3.54 to 3.66) | <0.001 |
| Republic of Korea | 1387.7 (747.91-2179.96) | 53.23 (28.75-84.36) | 15080.43 (9470.05-22620.44) | 126.41 (79.09-190.14) | 2.82 (2.73 to 2.91) | <0.001 |
| Republic of Moldova | 14.26 (8.68-21.8) | 2.78 (1.67-4.3) | 70.99 (38.66-122.13) | 8.92 (4.84-15.36) | 3.98 (3.81 to 4.15) | <0.001 |
| Romania | 439.59 (248.03-680.89) | 13.36 (7.44-20.89) | 969.09 (490.39-1609.58) | 18.4 (9.31-30.54) | 1.07 (0.8 to 1.27) | <0.001 |
| Russian Federation | 1986.71 (1170.58-3154.95) | 8.89 (5.22-14.17) | 6750.7 (3671.85-11397.78) | 21.13 (11.44-35.83) | 2.84 (2.53 to 3.14) | <0.001 |
| Rwanda | 343.77 (166.27-614.01) | 147.86 (72-261.57) | 1234.66 (603.36-2201.98) | 226.99 (111.32-403.49) | 1.41 (1.39 to 1.43) | <0.001 |
| Saint Kitts and Nevis | 5.2 (3.43-7.58) | 99.03 (65.38-144.51) | 14.24 (9.04-21.55) | 198.58 (124.71-303.16) | 2.4 (2.08 to 2.65) | <0.001 |
| Saint Lucia | 10.68 (7.04-15.48) | 98.39 (64.75-143.35) | 48.24 (30.24-73.03) | 165.32 (103.43-250.68) | 1.85 (1.76 to 1.94) | <0.001 |
| Saint Vincent and the Grenadines | 5.66 (3.73-8.16) | 63.42 (41.76-92.1) | 20.36 (12.56-31.35) | 117.54 (72.11-181.91) | 2.06 (1.94 to 2.17) | <0.001 |
| Samoa | 5.68 (3.67-8.55) | 66.99 (42.96-101.46) | 15.64 (9.46-24.43) | 105.86 (63.55-165.98) | 1.51 (1.46 to 1.56) | <0.001 |
| San Marino | 1.41 (0.88-2.12) | 29.43 (18.44-44.08) | 6.86 (4.01-10.88) | 53.74 (32.08-83.84) | 2.04 (2 to 2.09) | <0.001 |
| Sao Tome and Principe | 19.61 (9.85-33.88) | 281.43 (141.32-485.1) | 38.88 (20.21-66.06) | 390.8 (202.47-663.53) | 1.08 (1.06 to 1.1) | <0.001 |
| Saudi Arabia | 500.37 (340.27-705.85) | 92.92 (62.9-132.23) | 2673.1 (1782.59-3883.6) | 208.15 (136.95-305.71) | 2.63 (2.61 to 2.66) | <0.001 |
| Senegal | 779.2 (402.13-1328.58) | 254.47 (130.98-430.96) | 2308.85 (1214.26-3911.98) | 310.63 (163.52-524.51) | 0.61 (0.58 to 0.63) | <0.001 |
| Serbia | 263.1 (141.04-416.89) | 20.34 (10.51-32.81) | 767.53 (372.95-1265.37) | 33.49 (16.34-55.11) | 1.82 (1.62 to 2.09) | <0.001 |
| Seychelles | 3.25 (2.11-4.72) | 45.52 (29.6-66.26) | 14.96 (9.12-23.3) | 119.8 (72.64-187.13) | 3.24 (3.17 to 3.3) | <0.001 |
| Sierra Leone | 360.38 (180.01-629.08) | 171.09 (85.81-297.97) | 703.87 (361.34-1212.93) | 202.68 (104.3-347.96) | 0.55 (0.53 to 0.58) | <0.001 |
| Singapore | 57.07 (28.3-94.43) | 28.86 (14.51-47.74) | 1003.92 (545.47-1693.24) | 95.67 (51.84-161.42) | 3.89 (3.79 to 3.97) | <0.001 |
| Slovakia | 191.23 (123.62-280.07) | 25.35 (16.34-37.35) | 504.09 (301.46-815.06) | 39.26 (23.4-63.55) | 1.24 (0.92 to 1.47) | <0.001 |
| Slovenia | 45.86 (24.16-72.92) | 15.14 (7.94-24.17) | 223.9 (118.42-364.51) | 35.09 (18.74-56.75) | 2.81 (2.65 to 2.94) | <0.001 |
| Solomon Islands | 3.55 (2.21-5.47) | 32.77 (20.13-50.82) | 11.86 (6.74-18.97) | 41.5 (23.29-67) | 0.76 (0.72 to 0.8) | <0.001 |
| Somalia | 234.4 (113.05-430.61) | 151.86 (73.06-275.92) | 834.6 (409.7-1490.16) | 197.4 (96.88-349.18) | 0.86 (0.85 to 0.87) | <0.001 |
| South Africa | 3574.93 (1841.62-6150.04) | 167.42 (86.25-286.95) | 11237.32 (5806.82-19241.73) | 239.85 (123.8-407.98) | 1.16 (1.14 to 1.18) | <0.001 |
| South Sudan | 454.4 (217.36-815.58) | 182.21 (87.4-324.94) | 841.66 (412.77-1501.26) | 277.71 (136.35-491.4) | 1.4 (1.38 to 1.43) | <0.001 |
| Spain | 4006.5 (2461.89-6076.69) | 58.01 (35.58-88.11) | 14838.45 (9706.68-21611.49) | 93.48 (61.49-135.69) | 1.61 (1.54 to 1.69) | <0.001 |
| Sri Lanka | 704.24 (475.6-1013.38) | 68.32 (45.62-99.29) | 3152.29 (2047.05-4658.43) | 97.19 (62.72-144.29) | 1.19 (1.11 to 1.25) | <0.001 |
| Sudan | 350.94 (231.9-502.49) | 37.94 (24.94-54.87) | 1261.36 (791.33-1914.79) | 68.75 (42.94-104.62) | 1.98 (1.95 to 2.01) | <0.001 |
| Suriname | 24.85 (16.26-35.85) | 89.95 (58.78-129.63) | 134.55 (84.16-203.76) | 184.65 (115.46-279.69) | 2.39 (2.34 to 2.43) | <0.001 |
| Sweden | 688.78 (387.21-1108.22) | 31.72 (17.85-51.03) | 4364.34 (2256.2-7380.94) | 128.37 (66.9-216.55) | 4.95 (4.78 to 5.14) | <0.001 |
| Switzerland | 312.26 (168.44-493.97) | 21.73 (11.8-34.4) | 1827.08 (1004.65-2982.09) | 63.5 (35.61-102.18) | 3.7 (3.56 to 3.84) | <0.001 |
| Syrian Arab Republic | 377.75 (254.76-541.88) | 73.72 (49.36-106.28) | 1364.73 (871.63-2053.71) | 105.98 (66.87-160.83) | 1.18 (1.15 to 1.2) | <0.001 |
| Taiwan (Province of China) | 2028.26 (1355.08-2918.96) | 127.48 (84.39-185.85) | 15218.31 (10034.74-22443.11) | 272.84 (180.1-401.56) | 2.5 (2.46 to 2.56) | <0.001 |
| Tajikistan | 6.62 (3.87-10.48) | 2.21 (1.28-3.51) | 23.32 (13.11-38.26) | 4.19 (2.31-6.95) | 2.15 (2.08 to 2.21) | <0.001 |
| Thailand | 5167.08 (3486.11-7471.93) | 151.42 (101.61-219.7) | 48795.39 (32322.76-71326.07) | 355.63 (235.46-519.52) | 2.78 (2.74 to 2.82) | <0.001 |
| Timor-Leste | 12.29 (8.06-17.91) | 58 (37.51-85.57) | 69.6 (43.41-105.01) | 73.76 (45.69-112.14) | 0.74 (0.71 to 0.77) | <0.001 |
| Togo | 185.82 (94.75-328.13) | 180.74 (91.99-317.4) | 630.97 (322.68-1092.56) | 212.04 (108.82-364.32) | 0.51 (0.49 to 0.53) | <0.001 |
| Tokelau | 0.08 (0.05-0.11) | 48.95 (31.31-72.8) | 0.19 (0.11-0.29) | 97.53 (58.44-152.88) | 2.31 (2.27 to 2.35) | <0.001 |
| Tonga | 3.99 (2.49-6.08) | 70.25 (43.49-107.5) | 11.55 (6.67-18.28) | 123.43 (71.09-195.75) | 1.82 (1.77 to 1.87) | <0.001 |
| Trinidad and Tobago | 56.66 (37.56-81.83) | 58.37 (38.5-84.88) | 460.48 (291.78-691.35) | 187.29 (118.64-281.39) | 3.95 (3.73 to 4.16) | <0.001 |
| Tunisia | 318.83 (213.75-457.82) | 61.78 (41.06-89.45) | 1684.7 (1076.63-2535.31) | 110.13 (70.06-166.28) | 1.89 (1.86 to 1.91) | <0.001 |
| Turkey | 3084.43 (2108.79-4348.83) | 88.81 (60.14-126.14) | 16287.32 (10786.62-23801.91) | 147.26 (97.15-215.68) | 1.66 (1.58 to 1.74) | <0.001 |
| Turkmenistan | 23.21 (13.83-35.62) | 11.13 (6.6-17.18) | 109.2 (63.6-170.28) | 26.63 (15.31-41.91) | 2.85 (2.62 to 3.07) | <0.001 |
| Tuvalu | 0.26 (0.17-0.39) | 41.36 (26.03-62.88) | 0.92 (0.55-1.43) | 83.72 (49.77-131.33) | 2.32 (2.27 to 2.35) | <0.001 |
| Uganda | 964.22 (466.46-1744.87) | 163.13 (78.94-293.77) | 3241.56 (1586.92-5798.81) | 252.43 (123.48-449.99) | 1.44 (1.4 to 1.47) | <0.001 |
| Ukraine | 19.44 (11.48-30.65) | 0.22 (0.13-0.34) | 387.99 (201.09-675.69) | 3.66 (1.9-6.39) | 10.18 (9.77 to 10.63) | <0.001 |
| United Arab Emirates | 14.72 (10-20.79) | 52.89 (35.62-75.7) | 243.47 (162.85-348.42) | 121.16 (78.38-178.47) | 2.76 (2.71 to 2.81) | <0.001 |
| United Kingdom | 1478.25 (815.52-2379.3) | 11.98 (6.62-19.32) | 6606.37 (3548.76-10844.92) | 33.21 (17.93-54.55) | 3.4 (3.3 to 3.48) | <0.001 |
| United Republic of Tanzania | 2156.09 (1045.59-3936.68) | 217.96 (105.85-395.13) | 6528.5 (3221.69-11682.27) | 274.38 (135.64-489.34) | 0.8 (0.73 to 0.87) | <0.001 |
| United States of America | 18334.83 (10007.95-29809.18) | 42.14 (23.05-68.46) | 124966.05 (69016.24-206178.66) | 154.14 (85.23-254.04) | 4.38 (4.31 to 4.45) | <0.001 |
| United States Virgin Islands | 4.79 (3.16-6.97) | 54.95 (36.08-80.47) | 28.15 (17.65-43.07) | 109.78 (68.71-168.35) | 2.24 (2.21 to 2.26) | <0.001 |
| Uruguay | 233.01 (138.22-364.65) | 46.44 (27.51-72.81) | 642.46 (321.85-1059.36) | 78.87 (40.14-129.22) | 1.9 (1.8 to 1.99) | <0.001 |
| Uzbekistan | 167.35 (99.3-260.5) | 13.23 (7.81-20.7) | 677.76 (354.76-1130.61) | 26.13 (13.37-44.05) | 2.18 (1.97 to 2.32) | <0.001 |
| Vanuatu | 1.99 (1.27-3.01) | 38.88 (24.56-58.93) | 9.95 (5.98-15.53) | 66.1 (39.47-103.55) | 1.74 (1.72 to 1.76) | <0.001 |
| Venezuela (Bolivarian Republic of) | 479.09 (316.43-697) | 46.62 (30.63-68.16) | 5561.67 (3599.51-8384.64) | 153.41 (99.23-230.79) | 4.03 (3.92 to 4.15) | <0.001 |
| Viet Nam | 3376.94 (2210.06-4956.62) | 75.4 (49.24-110.98) | 12024.42 (7548.57-18043.97) | 114.71 (71.55-173.1) | 1.37 (1.35 to 1.38) | <0.001 |
| Yemen | 131.64 (88.4-189.57) | 29.63 (19.71-42.92) | 517.98 (328.34-785.22) | 40.13 (25.21-61.26) | 1 (0.98 to 1.01) | <0.001 |
| Zambia | 390.3 (187.96-703.24) | 160.85 (77.51-289.31) | 1092.03 (528.47-1938.03) | 193.98 (94.26-343.23) | 0.63 (0.61 to 0.65) | <0.001 |
| Zimbabwe | 627.4 (319.24-1082.84) | 174.76 (88.59-300.43) | 1126.54 (559.82-1992.73) | 210.13 (105.39-367.96) | 0.55 (0.49 to 0.59) | <0.001 |

Abbreviations: AAPC, average annual percentage change
