# Supplementary material for: Burden of heart failure attributable to chronic kidney disease in older adults (1990–2021): an analysis from the global burden of disease study
Source: Front Public Health. 2025 Jun 18;13:1606719. doi: 10.3389/fpubh.2025.1606719 (PMC12213463; doi:10.3389/fpubh.2025.1606719)
Supplement: Supplementary file 4 [file Table_4.docx]

**Supplementary table S4** YLDs of heart failure attributable to chronic kidney disease among older adults and corresponding AAPCs from 1990 to 2021 at the country/territory level.

| Country and territory | Number,1990 | YLDs,1990 | Number,2021 | YLDs,2021 | AAPC,1990-2021 | P value |
| --- | --- | --- | --- | --- | --- | --- |
| Afghanistan | 46.48 (24.94-76.26) | 6.82 (3.62-11.33) | 71.41 (35.34-122.33) | 9.12 (4.54-15.62) | 0.96 (0.93 to 0.99) | <0.001 |
| Albania | 9.62 (4.84-15.54) | 4.32 (2.15-7.02) | 31.52 (15.52-55.53) | 5.47 (2.68-9.66) | 0.78 (0.74 to 0.81) | <0.001 |
| Algeria | 80.53 (42.62-131.3) | 6.62 (3.48-10.95) | 462.74 (235.73-797.45) | 12.51 (6.32-21.71) | 2.1 (2.05 to 2.14) | <0.001 |
| American Samoa | 0.25 (0.13-0.41) | 12.44 (6.3-20.93) | 1.41 (0.7-2.42) | 29.17 (14.48-50.25) | 2.86 (2.8 to 2.93) | <0.001 |
| Andorra | 0.46 (0.23-0.78) | 7.41 (3.72-12.79) | 2.19 (1.06-3.89) | 10.26 (4.94-18.12) | 1.06 (1.01 to 1.11) | <0.001 |
| Angola | 54.52 (22.63-108.77) | 20.04 (8.36-39.5) | 245.05 (103.23-492.46) | 28.37 (12.22-56.44) | 1.14 (1.12 to 1.16) | <0.001 |
| Antigua and Barbuda | 0.99 (0.55-1.62) | 13.46 (7.47-21.96) | 2.61 (1.34-4.4) | 21.45 (10.98-36.34) | 1.59 (1.42 to 1.74) | <0.001 |
| Argentina | 338 (159.58-581.05) | 8.8 (4.17-15.19) | 1002.22 (457.99-1813.29) | 13.52 (6.2-24.42) | 1.31 (1.23 to 1.38) | <0.001 |
| Armenia | 0.72 (0.38-1.18) | 0.25 (0.13-0.41) | 27.64 (14.37-46.77) | 4.88 (2.53-8.26) | 10.44 (10.15 to 10.75) | <0.001 |
| Australia | 163.51 (82.36-278.33) | 6.67 (3.36-11.37) | 1051.36 (530.94-1854.44) | 16.14 (8.17-28.42) | 3.18 (3 to 3.32) | <0.001 |
| Austria | 63.95 (33.51-104.45) | 4.04 (2.13-6.6) | 321.9 (159.53-558.34) | 11.3 (5.62-19.58) | 3.41 (3.25 to 3.56) | <0.001 |
| Azerbaijan | 6.07 (3.07-10.28) | 1.17 (0.59-1.99) | 27.96 (13.46-51.89) | 2.67 (1.27-5) | 2.75 (2.68 to 2.8) | <0.001 |
| Bahamas | 1.15 (0.59-1.95) | 6.9 (3.53-11.67) | 5.5 (2.72-9.72) | 12.6 (6.2-22.26) | 2 (1.84 to 2.14) | <0.001 |
| Bahrain | 1.17 (0.64-1.88) | 8.62 (4.7-14.05) | 15.37 (8.4-25.36) | 21.46 (11.56-35.79) | 2.99 (2.95 to 3.02) | <0.001 |
| Bangladesh | 175.25 (95.84-284.52) | 3.79 (2.06-6.17) | 794.75 (412.29-1380.11) | 5.33 (2.74-9.29) | 1.07 (1.03 to 1.1) | <0.001 |
| Barbados | 4.92 (2.62-8.21) | 11.82 (6.33-19.7) | 16.2 (8.46-27.25) | 23.71 (12.4-39.9) | 2.33 (2.26 to 2.42) | <0.001 |
| Belarus | 3.01 (1.58-4.9) | 0.19 (0.1-0.31) | 18.51 (9.45-32.63) | 0.86 (0.44-1.52) | 5.34 (5.11 to 5.56) | <0.001 |
| Belgium | 84.77 (42.18-143.16) | 4.17 (2.07-7.06) | 272.68 (142.17-466.79) | 7.27 (3.81-12.38) | 1.75 (1.5 to 1.95) | <0.001 |
| Belize | 1.63 (0.87-2.71) | 14.43 (7.68-23.92) | 8.16 (4.16-13.93) | 26 (13.26-44.44) | 2.04 (1.95 to 2.13) | <0.001 |
| Benin | 47.91 (21.2-94.95) | 23.07 (10.29-45.32) | 144.33 (61.36-280.05) | 30.61 (13.22-59.01) | 0.92 (0.9 to 0.94) | <0.001 |
| Bermuda | 0.56 (0.29-0.93) | 7.49 (3.93-12.49) | 3.64 (1.87-6.25) | 19.18 (9.84-32.94) | 3.07 (3 to 3.13) | <0.001 |
| Bhutan | 1.08 (0.59-1.75) | 5.22 (2.83-8.53) | 6.01 (3.18-10.03) | 8.84 (4.66-14.8) | 1.71 (1.69 to 1.74) | <0.001 |
| Bolivia (Plurinational State of) | 55.41 (29.2-92.93) | 16.92 (8.98-28.42) | 356.59 (181.34-597.48) | 35.53 (18.2-59.54) | 2.42 (2.39 to 2.44) | <0.001 |
| Bosnia and Herzegovina | 13.48 (6.79-22.65) | 3.23 (1.6-5.49) | 41.5 (19.51-74.32) | 4.94 (2.31-8.89) | 1.4 (1.37 to 1.43) | <0.001 |
| Botswana | 7.33 (3.06-14.68) | 15.7 (6.7-31.01) | 34.19 (14.61-67.66) | 26.82 (11.6-52.78) | 1.74 (1.71 to 1.76) | <0.001 |
| Brazil | 618.09 (322.47-1072.7) | 6.9 (3.6-11.96) | 4566.45 (2238.95-8332.76) | 15.07 (7.4-27.47) | 2.58 (2.46 to 2.67) | <0.001 |
| Brunei Darussalam | 0.5 (0.15-0.97) | 5.78 (1.81-11.29) | 2.44 (0.69-4.82) | 8.83 (2.7-17.63) | 1.36 (1.33 to 1.38) | <0.001 |
| Bulgaria | 18.94 (9.84-30.69) | 1.28 (0.66-2.1) | 56.09 (27.25-100.64) | 2.76 (1.34-4.96) | 2.64 (2.51 to 2.74) | <0.001 |
| Burkina Faso | 116.4 (50.62-226.82) | 29.61 (13.09-57.14) | 292.8 (127.07-565.5) | 34.02 (14.93-65.18) | 0.46 (0.44 to 0.49) | <0.001 |
| Burundi | 37.79 (15.49-76.79) | 17.16 (7.11-34.67) | 101.78 (42.29-205.83) | 25.48 (10.71-51.11) | 1.31 (1.29 to 1.33) | <0.001 |
| Cabo Verde | 6.22 (2.69-11.96) | 20.31 (8.74-39.1) | 17.14 (7.64-32.67) | 35.3 (15.73-67.49) | 1.81 (1.79 to 1.83) | <0.001 |
| Cambodia | 20.43 (11.1-33.27) | 4.99 (2.69-8.18) | 92.4 (47.12-156.32) | 7.64 (3.85-13.05) | 1.39 (1.35 to 1.41) | <0.001 |
| Cameroon | 128.65 (55.14-253.36) | 33.53 (14.55-65.59) | 436.88 (196.33-832.67) | 41.29 (18.82-77.87) | 0.67 (0.62 to 0.72) | <0.001 |
| Canada | 239.29 (121.44-408.93) | 5.7 (2.89-9.75) | 1867.91 (896.7-3361.86) | 18.05 (8.67-32.42) | 3.73 (3.6 to 3.85) | <0.001 |
| Central African Republic | 15.66 (6.47-31.3) | 19.97 (8.36-39.17) | 31.86 (13.56-62.82) | 22.89 (9.95-44.42) | 0.45 (0.43 to 0.46) | <0.001 |
| Chad | 56.74 (24.45-112.35) | 19.39 (8.43-38.07) | 111.47 (46.37-217.57) | 22.45 (9.53-43.66) | 0.47 (0.45 to 0.48) | <0.001 |
| Chile | 79.38 (37.98-137.99) | 7.14 (3.42-12.46) | 561.89 (261.63-1021.93) | 16.86 (7.84-30.67) | 2.91 (2.8 to 3) | <0.001 |
| China | 4703.55 (2486.71-8205.82) | 5.44 (2.86-9.5) | 22411.63 (11179.08-40482.25) | 8.44 (4.2-15.25) | 1.48 (1.41 to 1.54) | <0.001 |
| Colombia | 140.94 (74.37-231.16) | 7.8 (4.11-12.83) | 1042.66 (531.78-1792.73) | 14.9 (7.59-25.56) | 2.14 (2.09 to 2.19) | <0.001 |
| Comoros | 3.89 (1.58-7.88) | 22.63 (9.36-45.31) | 15.86 (6.72-31.21) | 33.23 (14.23-65.08) | 1.26 (1.25 to 1.28) | <0.001 |
| Congo | 21.88 (9.13-43.02) | 25.65 (11.01-49.55) | 69.44 (30.05-134.05) | 34.16 (14.91-65.48) | 0.94 (0.93 to 0.95) | <0.001 |
| Cook Islands | 0.05 (0.02-0.08) | 3.77 (1.97-6.53) | 0.27 (0.13-0.5) | 8.38 (4.01-15.45) | 2.59 (2.54 to 2.63) | <0.001 |
| Costa Rica | 18.82 (9.89-30.68) | 9.31 (4.89-15.19) | 207.32 (104.37-350.66) | 30.05 (15.14-50.68) | 3.96 (3.91 to 4.03) | <0.001 |
| Coted'Ivoire | 76.37 (32.94-150.65) | 26.76 (11.65-52.29) | 289.22 (125.84-559.16) | 32.43 (14.34-62.14) | 0.62 (0.6 to 0.65) | <0.001 |
| Croatia | 15.38 (7.33-26) | 2.19 (1.03-3.74) | 67.82 (30.33-124.47) | 5.3 (2.37-9.72) | 2.78 (2.55 to 2.95) | <0.001 |
| Cuba | 56.48 (30.69-91.06) | 4.41 (2.39-7.14) | 302.26 (156.22-515.81) | 11.8 (6.12-20.06) | 3.26 (3.17 to 3.33) | <0.001 |
| Cyprus | 4.88 (2.17-8.58) | 5.84 (2.63-10.3) | 24.56 (11.49-43.91) | 9.36 (4.38-16.81) | 1.78 (1.4 to 2.14) | <0.001 |
| Czechia | 55.6 (28.16-90.98) | 3.12 (1.57-5.13) | 188.73 (87.57-337.32) | 6.13 (2.86-10.92) | 2.24 (1.9 to 2.54) | <0.001 |
| Democratic People's Republic of Korea | 72.96 (40.08-114.36) | 4.66 (2.54-7.42) | 220.21 (113.59-368.73) | 5.81 (2.98-9.74) | 0.72 (0.71 to 0.74) | <0.001 |
| Democratic Republic of the Congo | 299.85 (123.99-609.89) | 26.08 (11.07-51.78) | 810.29 (335.47-1593.41) | 28.88 (12.27-56.12) | 0.33 (0.3 to 0.36) | <0.001 |
| Denmark | 18.56 (9.05-31.65) | 1.66 (0.81-2.84) | 189.11 (86.77-340.68) | 10.75 (4.98-19.24) | 6.42 (6.27 to 6.57) | <0.001 |
| Djibouti | 1.83 (0.75-3.68) | 19.29 (8.07-38.49) | 15.04 (6.14-30.5) | 31.35 (13.05-62.93) | 1.59 (1.57 to 1.61) | <0.001 |
| Dominica | 0.73 (0.38-1.23) | 9.37 (4.91-15.86) | 1.68 (0.83-2.94) | 16.75 (8.3-29.39) | 1.93 (1.89 to 1.97) | <0.001 |
| Dominican Republic | 32.45 (17-53.24) | 8.34 (4.37-13.73) | 162.49 (77.84-290.99) | 13.64 (6.53-24.45) | 1.54 (1.42 to 1.62) | <0.001 |
| Ecuador | 81.07 (41.54-135.75) | 13.96 (7.15-23.45) | 683.83 (346.79-1172.66) | 34.47 (17.5-59.18) | 3 (2.94 to 3.07) | <0.001 |
| Egypt | 197.53 (105.89-322.59) | 8.84 (4.71-14.53) | 821.01 (414.78-1443.25) | 15.5 (7.77-27.37) | 1.84 (1.77 to 1.91) | <0.001 |
| El Salvador | 57.75 (31.32-93.51) | 16.58 (9-26.83) | 424.12 (227.72-702.59) | 53.12 (28.5-87.73) | 3.91 (3.81 to 4.02) | <0.001 |
| Equatorial Guinea | 3.24 (1.36-6.52) | 21.24 (9.09-42.03) | 17.77 (7.81-34.09) | 43.27 (19.24-82.36) | 2.36 (2.3 to 2.42) | <0.001 |
| Eritrea | 9.93 (4.05-20.23) | 15.11 (6.27-30.36) | 47.04 (18.92-95.53) | 23.23 (9.55-46.7) | 1.46 (1.43 to 1.5) | <0.001 |
| Estonia | 3.52 (1.85-5.91) | 1.36 (0.72-2.29) | 31.17 (12.87-62.13) | 7.8 (3.24-15.48) | 5.1 (4.79 to 5.39) | <0.001 |
| Eswatini | 5.27 (2.19-10.58) | 21.86 (9.22-43.26) | 13.84 (5.57-27.21) | 30.29 (12.65-59.04) | 1.04 (1.01 to 1.06) | <0.001 |
| Ethiopia | 443.62 (193.08-867.66) | 28.39 (12.74-54.66) | 1627.71 (738.21-3124.03) | 39.62 (18.12-75.42) | 1.09 (1.07 to 1.1) | <0.001 |
| Fiji | 1.88 (1-3.11) | 6.31 (3.31-10.49) | 9.33 (4.73-15.96) | 12.92 (6.5-22.17) | 2.31 (2.29 to 2.33) | <0.001 |
| Finland | 11.5 (5.48-19.75) | 1.27 (0.6-2.19) | 81.75 (37.44-151.87) | 4 (1.85-7.38) | 3.87 (3.61 to 4.14) | <0.001 |
| France | 1165.72 (619.25-1906.05) | 10.2 (5.43-16.7) | 5404.76 (2881.4-9219.71) | 23.33 (12.43-39.57) | 2.63 (2.45 to 2.81) | <0.001 |
| Gabon | 16 (6.85-30.64) | 27.88 (12.04-53.12) | 39.95 (17.26-77.7) | 44.11 (19.31-85.33) | 1.51 (1.49 to 1.53) | <0.001 |
| Gambia | 7.41 (3.07-14.78) | 24.27 (10.28-47.58) | 28.5 (12.29-54.74) | 30.65 (13.32-58.66) | 0.76 (0.74 to 0.78) | <0.001 |
| Georgia | 4.36 (2.18-7.41) | 0.57 (0.29-0.98) | 20.86 (6.99-40.18) | 2.57 (0.85-4.94) | 4.94 (4.65 to 5.24) | <0.001 |
| Germany | 589.52 (297.94-977.2) | 3.49 (1.76-5.79) | 3719.92 (1782.77-6774.83) | 11.94 (5.69-21.82) | 4.2 (3.97 to 4.42) | <0.001 |
| Ghana | 59.43 (25.01-120.81) | 11.53 (4.87-23.37) | 283.01 (115.72-575.12) | 19.61 (8.1-39.54) | 1.76 (1.74 to 1.77) | <0.001 |
| Greece | 155.02 (78.52-264.23) | 8.32 (4.24-14.23) | 306.01 (142.9-554.8) | 7.89 (3.71-14.32) | -0.02 (-0.24 to 0.19) | 0.855 |
| Greenland | 0.11 (0.04-0.2) | 4.17 (1.61-7.79) | 0.57 (0.22-1.12) | 8.7 (3.24-17.23) | 2.49 (2.4 to 2.58) | <0.001 |
| Grenada | 1.26 (0.65-2.09) | 12.35 (6.39-20.48) | 2.95 (1.46-5.1) | 22.94 (11.37-39.79) | 2.26 (2.15 to 2.39) | <0.001 |
| Guam | 0.47 (0.24-0.81) | 7.31 (3.75-12.68) | 4.53 (2.15-8.24) | 16.24 (7.77-29.4) | 2.6 (2.55 to 2.64) | <0.001 |
| Guatemala | 68.42 (34.85-116.78) | 20.02 (10.25-34.25) | 585.23 (303.37-976.52) | 45.06 (23.35-75.37) | 2.65 (2.58 to 2.72) | <0.001 |
| Guinea | 77.88 (32.43-152.53) | 23.01 (9.72-44.84) | 141.22 (60.55-271.53) | 26.03 (11.31-49.76) | 0.4 (0.38 to 0.43) | <0.001 |
| Guinea-Bissau | 7.33 (3.14-14.68) | 22.68 (9.98-44.37) | 13.69 (5.86-26.38) | 25.77 (11.24-49.24) | 0.39 (0.36 to 0.42) | <0.001 |
| Guyana | 2.12 (1.09-3.51) | 5.62 (2.87-9.33) | 9.06 (4.58-16.05) | 13.6 (6.84-24.12) | 3.12 (2.97 to 3.3) | <0.001 |
| Haiti | 18.44 (9.55-30.69) | 6.08 (3.13-10.18) | 57.11 (29.11-95.96) | 8.72 (4.42-14.77) | 1.18 (1.16 to 1.2) | <0.001 |
| Honduras | 13.55 (7.34-21.62) | 6.21 (3.33-9.97) | 68.49 (34.56-114.63) | 9.8 (4.96-16.51) | 1.5 (1.45 to 1.54) | <0.001 |
| Hungary | 23.82 (11.17-41.29) | 1.28 (0.6-2.24) | 110.34 (48.96-207.2) | 3.9 (1.74-7.31) | 3.64 (3.53 to 3.72) | <0.001 |
| Iceland | 0.34 (0.15-0.6) | 0.88 (0.38-1.53) | 2.52 (1.14-4.71) | 3.01 (1.37-5.58) | 3.51 (2.5 to 4.57) | <0.001 |
| India | 1713.45 (897.64-2985.67) | 4.3 (2.25-7.49) | 7940.79 (3824.71-14662.16) | 6.27 (3.02-11.56) | 1.24 (1.21 to 1.28) | <0.001 |
| Indonesia | 495.31 (257.6-849.79) | 5.51 (2.87-9.47) | 1684.04 (825.79-3057.73) | 7.24 (3.56-13.09) | 0.92 (0.86 to 1) | <0.001 |
| Iran (Islamic Republic of) | 159.75 (82.04-281.04) | 6.71 (3.44-11.77) | 964.07 (462.64-1794.89) | 11.61 (5.56-21.62) | 1.9 (1.84 to 1.97) | <0.001 |
| Iraq | 139.43 (75.77-223.77) | 16.03 (8.71-25.75) | 427.43 (223.16-717.91) | 19.45 (10.16-32.81) | 0.64 (0.6 to 0.67) | <0.001 |
| Ireland | 13.46 (6.67-22.78) | 2.69 (1.34-4.59) | 90.22 (43.7-161.67) | 8.3 (4.03-14.86) | 3.8 (3.66 to 3.89) | <0.001 |
| Israel | 47.36 (22.85-81.5) | 8.18 (3.98-14.03) | 415.99 (204.25-728.11) | 23.66 (11.6-41.4) | 3.79 (3.62 to 3.97) | <0.001 |
| Italy | 484.46 (227.25-872.08) | 4.2 (1.98-7.54) | 2131.52 (1028.3-3966.42) | 9.32 (4.49-17.36) | 2.73 (2.57 to 2.93) | <0.001 |
| Jamaica | 30.48 (15.85-50.59) | 12.77 (6.66-21.17) | 67.88 (34.68-116.61) | 16.81 (8.59-28.73) | 0.99 (0.9 to 1.07) | <0.001 |
| Japan | 1174.95 (516.72-2146.07) | 6.02 (2.68-10.99) | 7156.7 (3378.86-12988.64) | 10.79 (5.1-19.54) | 1.93 (1.89 to 1.97) | <0.001 |
| Jordan | 11.36 (6.29-18.48) | 9.96 (5.48-16.39) | 117.41 (60.27-200.75) | 17.68 (9.08-30.5) | 1.89 (1.84 to 1.93) | <0.001 |
| Kazakhstan | 14.96 (7.74-24.37) | 1.03 (0.53-1.68) | 72.53 (35.84-125.55) | 3.71 (1.8-6.45) | 4.32 (4.07 to 4.52) | <0.001 |
| Kenya | 143.35 (63.93-278.39) | 18.38 (8.24-35.55) | 495.66 (220.07-955.26) | 24.4 (10.94-46.64) | 1 (0.92 to 1.09) | <0.001 |
| Kiribati | 0.29 (0.14-0.5) | 8.9 (4.35-15.44) | 0.82 (0.4-1.49) | 13.85 (6.67-25.04) | 1.44 (1.39 to 1.5) | <0.001 |
| Kuwait | 6.31 (3.3-10.58) | 13.46 (6.98-22.63) | 35.35 (18.15-60.06) | 14.38 (7.3-24.55) | 0.28 (0.18 to 0.38) | <0.001 |
| Kyrgyzstan | 2.48 (1.24-4.28) | 0.72 (0.36-1.26) | 14.93 (7.87-24.79) | 2.95 (1.53-4.93) | 4.68 (4.6 to 4.77) | <0.001 |
| Lao People's Democratic Republic | 14.01 (7.45-23.39) | 7.33 (3.87-12.26) | 48.93 (24.71-84.32) | 11.13 (5.56-19.23) | 1.38 (1.36 to 1.39) | <0.001 |
| Latvia | 3.01 (1.58-5.02) | 0.67 (0.35-1.12) | 18.61 (8.58-34.04) | 3.23 (1.5-5.89) | 5.85 (5.58 to 6.12) | <0.001 |
| Lebanon | 27.01 (14.66-43.18) | 12.25 (6.63-19.76) | 230.89 (127.46-375.68) | 28.85 (15.91-46.93) | 2.86 (2.78 to 2.93) | <0.001 |
| Lesotho | 12.97 (5.22-26.01) | 15.02 (6.12-29.77) | 22.24 (9.21-44.28) | 23.01 (9.84-45.13) | 1.39 (1.37 to 1.41) | <0.001 |
| Liberia | 33.26 (14.36-65.44) | 29.01 (12.67-56.34) | 61.76 (27.11-118.49) | 35.7 (15.85-68.17) | 0.68 (0.63 to 0.72) | <0.001 |
| Libya | 25.62 (13.89-41.57) | 12.93 (6.98-21.04) | 92 (46.67-159.39) | 18.69 (9.47-32.4) | 1.17 (1.11 to 1.22) | <0.001 |
| Lithuania | 3.32 (1.73-5.5) | 0.59 (0.31-0.98) | 24.49 (11.52-44.59) | 3.06 (1.44-5.56) | 5.2 (4.92 to 5.41) | <0.001 |
| Luxembourg | 2.79 (1.22-4.88) | 4.2 (1.86-7.36) | 19.88 (9.42-36.15) | 13.28 (6.29-24.14) | 3.71 (3.49 to 3.88) | <0.001 |
| Madagascar | 60.67 (24.52-125.65) | 13.17 (5.37-27.09) | 141.01 (57.97-290.64) | 17 (7.15-34.66) | 0.85 (0.83 to 0.87) | <0.001 |
| Malawi | 71.56 (29.09-146.89) | 21.65 (9.02-43.86) | 190.73 (78.86-383.77) | 29.08 (12.19-57.62) | 0.96 (0.95 to 0.97) | <0.001 |
| Malaysia | 85.87 (45.6-140.15) | 8.92 (4.73-14.57) | 526.11 (265.54-871.77) | 16.88 (8.57-28) | 2.06 (2.02 to 2.09) | <0.001 |
| Maldives | 1.1 (0.58-1.83) | 15.39 (8.1-25.88) | 7.46 (3.95-12.62) | 23.39 (12.36-39.73) | 1.32 (1.28 to 1.35) | <0.001 |
| Mali | 89.08 (37.48-177.3) | 27.08 (11.65-53.28) | 243.46 (103-476.43) | 32.51 (14.07-63.04) | 0.59 (0.57 to 0.61) | <0.001 |
| Malta | 2.14 (1.1-3.58) | 4.4 (2.26-7.41) | 15.6 (7.54-28.64) | 10.66 (5.18-19.52) | 2.79 (2.58 to 2.92) | <0.001 |
| Marshall Islands | 0.11 (0.05-0.18) | 6.88 (3.55-11.73) | 0.35 (0.18-0.61) | 12.89 (6.51-22.61) | 2.09 (2.06 to 2.13) | <0.001 |
| Mauritania | 27.65 (11.69-53.32) | 27.52 (11.84-52.7) | 76.62 (33.53-147.27) | 35.45 (15.61-67.88) | 0.79 (0.76 to 0.83) | <0.001 |
| Mauritius | 9.29 (4.6-15.7) | 12.42 (6.13-21.03) | 84.52 (44.36-140.97) | 36.86 (19.4-61.4) | 3.58 (3.51 to 3.65) | <0.001 |
| Mexico | 1016.51 (551.98-1689.4) | 22.84 (12.37-38.03) | 6564.9 (3451.23-11281.33) | 43.83 (22.99-75.4) | 2.17 (2.1 to 2.25) | <0.001 |
| Micronesia (Federated States of) | 0.33 (0.17-0.56) | 6.65 (3.39-11.33) | 0.84 (0.41-1.46) | 13.42 (6.5-23.52) | 2.32 (2.27 to 2.36) | <0.001 |
| Monaco | 0.36 (0.19-0.6) | 3.39 (1.77-5.67) | 1.32 (0.63-2.35) | 8.61 (4.16-15.21) | 3.1 (2.98 to 3.2) | <0.001 |
| Mongolia | 3.61 (1.72-6.12) | 3.06 (1.46-5.21) | 9.46 (4.24-17.17) | 4.52 (1.98-8.32) | 1.24 (1.15 to 1.3) | <0.001 |
| Montenegro | 3.83 (1.92-6.44) | 5.34 (2.67-8.99) | 8.26 (4.14-14.21) | 6.55 (3.25-11.34) | 0.68 (0.64 to 0.71) | <0.001 |
| Morocco | 236.48 (126.58-387) | 15.73 (8.42-25.78) | 812.46 (421.51-1378.72) | 22.35 (11.61-37.98) | 1.13 (1.1 to 1.14) | <0.001 |
| Mozambique | 105.35 (46.76-204.21) | 22.1 (9.97-42.16) | 264.12 (116.57-500.39) | 30.57 (13.87-56.89) | 1.07 (1.05 to 1.08) | <0.001 |
| Myanmar | 103.5 (56.41-171.23) | 4.79 (2.59-8.01) | 380.22 (195.17-657.25) | 7.55 (3.85-13.12) | 1.48 (1.43 to 1.53) | <0.001 |
| Namibia | 8.28 (3.33-16.88) | 14.83 (6.18-29.88) | 26.16 (10.87-52) | 21.13 (8.88-41.67) | 1.15 (1.12 to 1.17) | <0.001 |
| Nauru | 0.02 (0.01-0.04) | 6.57 (3.35-11.13) | 0.05 (0.02-0.09) | 10.33 (4.95-18.51) | 1.44 (1.36 to 1.48) | <0.001 |
| Nepal | 53.2 (28.36-85.93) | 6.45 (3.44-10.53) | 257.94 (132.03-450.53) | 10.72 (5.49-18.7) | 1.62 (1.59 to 1.65) | <0.001 |
| Netherlands | 92.02 (48.11-156.71) | 3.51 (1.83-5.98) | 574.36 (277.89-1029.7) | 11.24 (5.45-20.11) | 4.02 (3.76 to 4.25) | <0.001 |
| New Zealand | 20.37 (9.82-36.34) | 4.08 (1.97-7.28) | 118.11 (55.07-219.61) | 10.17 (4.75-18.89) | 3.1 (2.95 to 3.25) | <0.001 |
| Nicaragua | 30.7 (16.22-49.64) | 18.61 (9.83-30.1) | 239.96 (129.62-394.13) | 43.07 (23.21-70.93) | 2.79 (2.75 to 2.83) | <0.001 |
| Niger | 45.62 (18.66-91.85) | 20.9 (8.7-41.31) | 160.25 (68.01-307.44) | 23.58 (10.21-44.87) | 0.39 (0.37 to 0.41) | <0.001 |
| Nigeria | 3088.66 (1545.82-5501.55) | 71.92 (36.36-127.35) | 7596.54 (3847.01-13357.75) | 95.14 (48.64-166.46) | 0.87 (0.81 to 0.91) | <0.001 |
| Niue | 0.02 (0.01-0.04) | 8.21 (4.14-14.07) | 0.04 (0.02-0.08) | 16.96 (8.27-29.93) | 2.36 (2.31 to 2.4) | <0.001 |
| North Macedonia | 5 (2.53-8.34) | 2.39 (1.21-4.01) | 15.17 (7.33-27.12) | 3.66 (1.75-6.6) | 1.42 (1.39 to 1.46) | <0.001 |
| Northern Mariana Islands | 0.22 (0.11-0.37) | 19.88 (10.31-33.31) | 1.51 (0.75-2.65) | 31.98 (15.88-55.91) | 1.49 (1.45 to 1.53) | <0.001 |
| Norway | 15.35 (5.63-29.59) | 1.57 (0.59-3.03) | 99.39 (41-192.96) | 6.68 (2.78-12.92) | 4.98 (4.85 to 5.11) | <0.001 |
| Oman | 3.31 (1.84-5.19) | 5.37 (2.96-8.49) | 21.3 (11.34-35.42) | 13.64 (7.26-22.82) | 3.08 (3.01 to 3.12) | <0.001 |
| Pakistan | 552.45 (283.66-965.06) | 9.63 (4.95-16.81) | 1408.17 (681.24-2597.8) | 12.79 (6.2-23.59) | 0.9 (0.88 to 0.92) | <0.001 |
| Palau | 0.13 (0.07-0.23) | 13.45 (6.84-22.95) | 0.5 (0.25-0.86) | 24.34 (12.3-41.96) | 1.98 (1.94 to 2.01) | <0.001 |
| Palestine | 9.65 (5.21-15.54) | 10.46 (5.64-16.94) | 36.47 (19.03-60.33) | 15.36 (8.02-25.6) | 1.24 (1.21 to 1.27) | <0.001 |
| Panama | 18.47 (9.68-30.68) | 10.72 (5.63-17.77) | 191.06 (100.52-320.22) | 34.32 (18.07-57.39) | 3.94 (3.88 to 4.02) | <0.001 |
| Papua New Guinea | 4.61 (2.48-7.74) | 3.21 (1.71-5.4) | 18.16 (9.38-31.94) | 4.52 (2.29-7.96) | 1.11 (1.07 to 1.14) | <0.001 |
| Paraguay | 22.27 (11.92-36.75) | 8.95 (4.81-14.78) | 106.73 (53.72-183.27) | 15.92 (8.03-27.24) | 1.95 (1.89 to 2) | <0.001 |
| Peru | 312.82 (168.48-507.16) | 23.78 (12.8-38.59) | 1900.01 (1005.99-3188.94) | 46.56 (24.66-77.98) | 2.23 (2.15 to 2.3) | <0.001 |
| Philippines | 260.79 (134.93-453.39) | 9.66 (5.01-16.75) | 1148.74 (553.66-2112.82) | 13.78 (6.64-25.3) | 1.15 (1.13 to 1.18) | <0.001 |
| Poland | 330.9 (173.62-574.26) | 6.2 (3.24-10.75) | 766.6 (387.36-1381.95) | 7.67 (3.87-13.84) | 1.27 (0.93 to 1.58) | <0.001 |
| Portugal | 108.84 (56.17-182.02) | 6.42 (3.33-10.73) | 595.98 (302.06-1046.26) | 15.14 (7.66-26.55) | 3.06 (2.95 to 3.17) | <0.001 |
| Puerto Rico | 84.46 (44.63-139) | 18.49 (9.81-30.46) | 424.84 (221.67-737.95) | 39.1 (20.51-67.56) | 2.43 (2.35 to 2.51) | <0.001 |
| Qatar | 0.55 (0.3-0.89) | 8.41 (4.49-13.78) | 13.66 (7.22-22.85) | 24.95 (13.07-42.33) | 3.56 (3.49 to 3.63) | <0.001 |
| Republic of Korea | 177.41 (81.98-296.2) | 6.73 (3.13-11.39) | 1901.44 (972.51-3214.79) | 15.92 (8.12-26.98) | 2.82 (2.73 to 2.89) | <0.001 |
| Republic of Moldova | 1.84 (0.95-3.11) | 0.36 (0.18-0.61) | 9.2 (4.33-17.52) | 1.16 (0.54-2.21) | 3.98 (3.81 to 4.15) | <0.001 |
| Romania | 56.77 (26.38-96.11) | 1.72 (0.8-2.94) | 125.34 (54.59-228.65) | 2.38 (1.04-4.34) | 0.98 (0.77 to 1.19) | <0.001 |
| Russian Federation | 257.07 (132.35-448.31) | 1.15 (0.59-2.01) | 872.25 (413.21-1607.38) | 2.73 (1.29-5.05) | 2.83 (2.54 to 3.12) | <0.001 |
| Rwanda | 42.95 (17.66-85.35) | 18.25 (7.66-35.97) | 154.28 (63.37-306.41) | 28.11 (11.75-55.79) | 1.41 (1.38 to 1.42) | <0.001 |
| Saint Kitts and Nevis | 0.65 (0.33-1.09) | 12.38 (6.34-20.61) | 1.79 (0.9-3.06) | 24.79 (12.55-42.49) | 2.39 (2.09 to 2.63) | <0.001 |
| Saint Lucia | 1.35 (0.67-2.27) | 12.37 (6.24-20.81) | 6.03 (3.16-10.4) | 20.65 (10.81-35.65) | 1.83 (1.74 to 1.92) | <0.001 |
| Saint Vincent and the Grenadines | 0.72 (0.37-1.18) | 8.02 (4.12-13.27) | 2.56 (1.25-4.44) | 14.76 (7.19-25.63) | 2.04 (1.93 to 2.14) | <0.001 |
| Samoa | 0.72 (0.37-1.23) | 8.41 (4.29-14.53) | 1.97 (0.95-3.42) | 13.23 (6.39-23.13) | 1.49 (1.44 to 1.53) | <0.001 |
| San Marino | 0.18 (0.09-0.3) | 3.75 (1.93-6.27) | 0.86 (0.43-1.55) | 6.8 (3.43-12.04) | 1.98 (1.91 to 2.05) | <0.001 |
| Sao Tome and Principe | 2.43 (1.06-4.78) | 34.75 (15.17-67.99) | 4.83 (2.19-9.32) | 48.37 (21.97-93) | 1.09 (1.07 to 1.11) | <0.001 |
| Saudi Arabia | 61.89 (33.28-100.66) | 11.4 (6.12-18.65) | 333.19 (177.66-541.57) | 25.63 (13.64-41.91) | 2.64 (2.61 to 2.67) | <0.001 |
| Senegal | 96.06 (42.28-181.52) | 31.18 (13.86-58.67) | 286.65 (127.57-545.54) | 38.37 (17.24-72.71) | 0.63 (0.6 to 0.66) | <0.001 |
| Serbia | 33.97 (15.24-58.75) | 2.62 (1.14-4.6) | 98.87 (40.43-177.07) | 4.31 (1.77-7.71) | 1.8 (1.61 to 2.06) | <0.001 |
| Seychelles | 0.41 (0.22-0.66) | 5.77 (3.1-9.21) | 1.86 (0.91-3.24) | 14.85 (7.21-25.91) | 3.19 (3.13 to 3.25) | <0.001 |
| Sierra Leone | 44.54 (18.86-88.44) | 21.02 (9.04-41.35) | 87.43 (38.16-172.22) | 25.02 (11.02-48.87) | 0.56 (0.54 to 0.59) | <0.001 |
| Singapore | 7.27 (3.13-12.87) | 3.65 (1.6-6.46) | 126.63 (57.81-235.85) | 12.05 (5.49-22.44) | 3.87 (3.78 to 3.95) | <0.001 |
| Slovakia | 24.67 (13.17-40.33) | 3.27 (1.74-5.37) | 64.76 (32.33-117.45) | 5.04 (2.51-9.14) | 1.25 (0.95 to 1.48) | <0.001 |
| Slovenia | 5.93 (2.72-10.4) | 1.96 (0.89-3.44) | 28.67 (12.97-52.21) | 4.5 (2.06-8.13) | 2.79 (2.63 to 2.9) | <0.001 |
| Solomon Islands | 0.46 (0.24-0.78) | 4.17 (2.13-7.13) | 1.52 (0.73-2.73) | 5.28 (2.5-9.54) | 0.75 (0.71 to 0.79) | <0.001 |
| Somalia | 29.12 (12.22-59.26) | 18.61 (7.87-37.64) | 103.6 (42.5-205.99) | 24.12 (10.1-47.39) | 0.85 (0.82 to 0.87) | <0.001 |
| South Africa | 445.5 (202.7-845.15) | 20.78 (9.49-39.3) | 1402.87 (647.89-2628.53) | 29.79 (13.83-55.57) | 1.16 (1.14 to 1.19) | <0.001 |
| South Sudan | 55.7 (22.33-112.57) | 22.08 (8.97-44.32) | 103.48 (43.64-201.31) | 33.84 (14.48-65.71) | 1.41 (1.39 to 1.43) | <0.001 |
| Spain | 506.01 (255.09-854.58) | 7.3 (3.7-12.33) | 1850.69 (1008.37-3065.73) | 11.73 (6.38-19.42) | 1.6 (1.52 to 1.68) | <0.001 |
| Sri Lanka | 88.57 (47.75-144.71) | 8.51 (4.58-13.98) | 393.97 (206.08-661.23) | 12.09 (6.31-20.33) | 1.18 (1.12 to 1.22) | <0.001 |
| Sudan | 44.97 (24.53-73.31) | 4.81 (2.6-7.92) | 159.13 (80.46-277.49) | 8.62 (4.34-15.07) | 1.94 (1.91 to 1.97) | <0.001 |
| Suriname | 3.13 (1.63-5.15) | 11.3 (5.87-18.64) | 16.79 (8.62-28.52) | 23 (11.84-39.07) | 2.33 (2.28 to 2.38) | <0.001 |
| Sweden | 87.95 (42.73-159.24) | 4.05 (1.97-7.33) | 549.43 (249.2-1024.33) | 16.21 (7.39-30.21) | 4.89 (4.72 to 5.09) | <0.001 |
| Switzerland | 39.93 (17.96-69.16) | 2.78 (1.26-4.81) | 229.83 (106.08-413.1) | 8.02 (3.75-14.27) | 3.66 (3.53 to 3.78) | <0.001 |
| Syrian Arab Republic | 47.46 (25.82-78.34) | 9.21 (4.97-15.28) | 171.65 (88.54-290.42) | 13.2 (6.82-22.41) | 1.16 (1.14 to 1.18) | <0.001 |
| Taiwan (Province of China) | 256.17 (133.13-423.85) | 16 (8.31-26.47) | 1903.03 (1012.95-3137.83) | 34.13 (18.19-56.07) | 2.5 (2.44 to 2.56) | <0.001 |
| Tajikistan | 0.86 (0.43-1.47) | 0.29 (0.14-0.49) | 3.02 (1.44-5.42) | 0.54 (0.25-0.97) | 2.15 (2.08 to 2.2) | <0.001 |
| Thailand | 641.35 (344.97-1061.83) | 18.69 (10.08-30.99) | 6029.78 (3314.76-9922.2) | 43.94 (24.18-72.23) | 2.8 (2.73 to 2.85) | <0.001 |
| Timor-Leste | 1.55 (0.81-2.55) | 7.24 (3.72-12.04) | 8.77 (4.45-14.99) | 9.2 (4.64-15.76) | 0.74 (0.71 to 0.77) | <0.001 |
| Togo | 23.11 (9.86-45.94) | 22.25 (9.65-43.94) | 78.71 (32.8-156.52) | 26.2 (11.18-51.34) | 0.52 (0.5 to 0.53) | <0.001 |
| Tokelau | 0.01 (0.01-0.02) | 6.19 (3.22-10.51) | 0.02 (0.01-0.04) | 12.12 (5.9-21.31) | 2.26 (2.21 to 2.31) | <0.001 |
| Tonga | 0.5 (0.23-0.9) | 8.74 (3.96-15.6) | 1.44 (0.67-2.62) | 15.36 (7.15-28.06) | 1.82 (1.78 to 1.87) | <0.001 |
| Trinidad and Tobago | 7.18 (3.65-11.96) | 7.36 (3.76-12.32) | 57.61 (29.74-97.94) | 23.4 (12.12-39.78) | 3.92 (3.69 to 4.14) | <0.001 |
| Tunisia | 40.46 (20.77-66.82) | 7.76 (4-12.87) | 210.99 (107.83-365.72) | 13.74 (7-23.83) | 1.86 (1.81 to 1.89) | <0.001 |
| Turkey | 388.11 (209.49-620.3) | 11.12 (5.96-17.91) | 2031.22 (1044.85-3374.99) | 18.32 (9.44-30.49) | 1.62 (1.56 to 1.68) | <0.001 |
| Turkmenistan | 3 (1.5-5.07) | 1.44 (0.72-2.44) | 14.1 (6.92-24.5) | 3.44 (1.67-6.02) | 2.84 (2.61 to 3.05) | <0.001 |
| Tuvalu | 0.03 (0.02-0.06) | 5.25 (2.75-9.01) | 0.12 (0.06-0.21) | 10.47 (5.07-18.9) | 2.28 (2.25 to 2.31) | <0.001 |
| Uganda | 120.3 (49.33-242.84) | 20.15 (8.36-40.26) | 403.75 (168.39-802.34) | 31.23 (13.11-61.77) | 1.45 (1.42 to 1.47) | <0.001 |
| Ukraine | 2.52 (1.28-4.46) | 0.03 (0.01-0.05) | 50.25 (23.16-94.37) | 0.47 (0.22-0.89) | 10.16 (9.75 to 10.62) | <0.001 |
| United Arab Emirates | 1.88 (1.02-3.01) | 6.68 (3.61-10.83) | 31.05 (16.71-51.14) | 15.12 (7.87-25.61) | 2.72 (2.67 to 2.77) | <0.001 |
| United Kingdom | 189.87 (92.34-336.85) | 1.54 (0.75-2.72) | 834.14 (402.75-1508.03) | 4.2 (2.03-7.61) | 3.35 (3.26 to 3.43) | <0.001 |
| United Republic of Tanzania | 267.23 (110.45-549.99) | 26.7 (11.23-54.23) | 809.31 (339-1597.62) | 33.81 (14.26-66.53) | 0.85 (0.75 to 0.95) | <0.001 |
| United States of America | 2347.8 (1116.15-4219.3) | 5.39 (2.57-9.68) | 15704.88 (7722.53-29082.43) | 19.38 (9.54-35.85) | 4.31 (4.25 to 4.38) | <0.001 |
| United States Virgin Islands | 0.61 (0.32-0.98) | 6.95 (3.6-11.33) | 3.54 (1.71-6.13) | 13.78 (6.68-23.8) | 2.22 (2.2 to 2.24) | <0.001 |
| Uruguay | 29.61 (14.23-51.28) | 5.89 (2.83-10.2) | 80.61 (35.25-149.01) | 9.93 (4.37-18.27) | 1.87 (1.79 to 1.96) | <0.001 |
| Uzbekistan | 21.62 (10.79-37.01) | 1.71 (0.85-2.94) | 87.54 (39.36-159.03) | 3.37 (1.48-6.21) | 2.18 (1.98 to 2.31) | <0.001 |
| Vanuatu | 0.26 (0.14-0.43) | 4.93 (2.57-8.34) | 1.26 (0.62-2.25) | 8.32 (4.01-14.8) | 1.72 (1.7 to 1.74) | <0.001 |
| Venezuela (Bolivarian Republic of) | 61.11 (32.86-99.74) | 5.93 (3.18-9.72) | 697.68 (354.35-1182.8) | 19.21 (9.77-32.47) | 3.98 (3.86 to 4.08) | <0.001 |
| Viet Nam | 424.13 (219.12-704.78) | 9.41 (4.85-15.66) | 1500.05 (758.76-2599.25) | 14.23 (7.18-24.71) | 1.36 (1.34 to 1.37) | <0.001 |
| Yemen | 16.92 (9.15-27.01) | 3.77 (2.03-6.09) | 66.4 (34.08-111.04) | 5.11 (2.6-8.62) | 1 (0.99 to 1.02) | <0.001 |
| Zambia | 48.56 (19.24-99.57) | 19.81 (7.94-40.41) | 135.68 (54.74-271.88) | 23.89 (9.89-47.64) | 0.61 (0.58 to 0.64) | <0.001 |
| Zimbabwe | 78.22 (34.1-154.3) | 21.59 (9.44-41.98) | 140.99 (59.07-282.15) | 26.03 (11.19-51.35) | 0.56 (0.5 to 0.6) | <0.001 |

Abbreviations: YLDs, years lived with disability; AAPC, average annual percentage change
